# ﻿*Hexatoma* crane flies (Diptera, Limoniidae) of Korea

**DOI:** 10.3897/zookeys.1105.82495

**Published:** 2022-06-15

**Authors:** Sigitas Podenas, Sun-Jae Park, Hye-Woo Byun, Virginija Podeniene

**Affiliations:** 1 Nature Research Centre, Akademijos str. 2, LT–08412 Vilnius, Lithuania Instute of Biosciences, Life Sciences Centre of Vilnius University Vilnius Lithuania; 2 Instute of Biosciences, Life Sciences Centre of Vilnius University, Sauletekio str. 7, LT–10257 Vilnius, Lithuania Nature Research Centre Vilnius Lithuania; 3 Animal Resources Division, National Institute of Biological Resources, Incheon 22689, Republic of Korea Animal Resources Division, National Institute of Biological Resources Incheon Republic of Korea

**Keywords:** East Palaearctic, Limnophilinae, North Korea, South Korea, Taiwan, taxonomy

## Abstract

*Hexatoma* Latreille, 1809 is a large genus of short-palped crane flies with a worldwide distribution. Accounting for more than 60 percent of global species, 362 extant species occur in Asia. Prior to our study, *Hexatoma* crane flies on the Korean Peninsula (both the Democratic People’s Republic of Korea and the Republic of Korea) had been studied for 90 years starting in 1930, but only three species had been recorded, with one of them being a misidentification. This study adds six species to the fauna of the Korean Peninsula, all of which belong to the subgenus H. (Eriocera) Macquart, 1838. General information on genus and subgenus morphological characters is presented in this paper, with a redescription of each species based on Korean specimens, as well as illustrations of both sexes, the elevation range, the period of activity, habitat information, general distribution, and a distribution map for the Korean Peninsula. Three species H. (E.) ilwola Podenas, **sp. nov.**, H. (E.) pianigra Podenas, **sp. nov.** and H. (E.) serenensis Podenas, **sp. nov.** are described as new to science. Hexatoma (E.) lygropis (Alexander, 1920) is deleted from the Korean species list as a misidentification. This publication is a continuation of our previous work on short-palped crane flies (Limoniidae) from Korea.

## ﻿Introduction

Investigations into Korean short-palped crane flies, family Limoniidae (Diptera), began more than a century ago with the first specimens collected as early as 1915 (Podenas et al. 2019). The first publication on that group of insects, with four descriptions of new species, was written by Ch. P. Alexander (Alexander 1934a). He described 49 species from Korea, most of them from the northern part of the peninsula, now the Democratic People’s Republic of Korea (North Korea) (Podenas 2013). Nearly eighty years later, in 2012, further investigations into the Limoniidae crane flies on the Korean Peninsula were initiated by the authors of this publication and researchers from the National Institute of Biological Resources in Incheon, the Republic of Korea (South Korea). Prior to those studies, 95 species of Limoniidae were recorded from the North and South Koreas (Podenas 2013), the new findings summarized in the National List of Species of Korea a few years later (Cho 2019), this already including 144 species.

The original description of the genus *Hexatoma* (Latreille 1809) was based on a male of a single species *H.nigra* Latreille, 1809. It listed just antennal and wing venation characters, such as a 6-segmented antenna, two short basal antennomeres, an especially short subglobular pedicel, four long cylindrical flagellomeres and an open discal cell with parallel veins reaching the wing margin. Recently, the genus has been divided into six subgenera. All of them have sexually dimorphic antennae, with the male antennae longer than that of female. In some species, the male antenna is several times longer than the body. Wing venation, especially a closed or open discal cell, is used for discrimination of subgenera. Male terminalia are comparatively simple, with a wide epandrium, usually elongate gonocoxite, two pairs of gonostyli and a simple aedeagus. On the other hand, in more tropical and subtropical regions, *Hexatoma* are distinguished on striking color patterns of the body and wings.

The genus has a worldwide distribution and includes 596 extant species (Oosterbroek 2022). *Hexatoma* is especially diverse in Asia, from where we know 362 species.

Larvae of all known Palaearctic *Hexatoma* species are aquatic, most of them developing in rivers with sandy or gravel bottoms, some Nearctic species developing in bogs (Alexander 1920) while the mating behavior includes swarming of males above the water surface observed during collecting trips in Asia.

The second publication on Korean crane flies (Alexander 1934b) included the original description of *H.masakii*, a species endemic to South Korea. These specimens were collected in 1930 by I. Tabashi. The last specimens of that species were collected in 1954 in an area close to the type locality. At the beginning of our studies, three species of *Hexatoma* had been recorded from the Korean Peninsula (Cho 2019), including another endemic species, H. (E.) pernigrina Alexander, 1938, that had been described from North Korea. Added in 1971 (Kim 1971), these three species also included *H.lygropis* (Alexander, 1920) that was a misidentification of *H.pernigrina*.

Commensing our studies, we expected a high diversity of *Hexatoma* crane flies in Korea due to the abundance of suitable habitats, specifically rivers with sandy or gravel bottoms.

Since 2012, crane flies have been collected annually in different localities, at different times and using different methods throughout the country. Despite original and subsequent descriptions of East Palearctic species over a long period of time, some of these species were known only from the original descriptions and no illustrations were available. The aim of our study was to document, redescribe, illustrate, and prepare keys for all Korean crane fly species identified to date. In this article, we provide photographs of important taxonomical details, such as antennae, wings and male and female terminalia. We also include distribution maps of the Korean species, as well as a key for all the species of the Korean Peninsula. This publication is a continuation of our previous work on short-palped crane flies (Limoniidae) from Korea. We plan similar treatments of the subfamily Chioneinae and the family Pediciidae which potentially could yield many more species for the Peninsula.

## ﻿Materials and methods

Crane flies available for this study (Table 1) are preserved in these scientific collections: the Hungarian Natural History Museum, Budapest, Hungary (**HNHM**), specimens collected in 1971 in North Korea by S. Horvatovich and J. Papp; Korea University, Seoul, South Korea (**KU**), specimens collected in 1973–2015 in South Korea by entomologists and students of Korea University; the National Institute of Biological Resources (**NIBR**), Incheon, South Korea, specimens collected in 2011–2019 in South Korea mostly by the authors of this publication; the Snow Entomological Museum, University of Kansas, Lawrence, KS, USA (**SMEK**), specimens collected in 1954 in South Korea by Dr. G. W. Byers; the United States National Museum, the Smithsonian Institution, Washington DC, USA (**USNM**), specimens collected in 1930–1940 in the central and northern parts of the Korean Peninsula (now North and South Koreas) by I. Tabashi and A. M. Yankovsky; the Natural History Museum, London, United Kingdom (**NHMUK**), comparative material; Naturalis biodiversity center, Leiden, Netherlands (**Naturalis**), comparative material; and Nature Research Center, Vilnius, Lithuania (**NRC**), comparative material.

Adult crane flies were collected in various ways, including by insect nets, with Malaise traps, LED light traps, black light traps, Mosquito Magnet traps (Pro Model, Woodstream Corp., Lititz, PA), New Jersey traps and at light sources. Some specimens were preserved dry in envelopes in the field and were later mounted at the laboratory on their side on a paper point, with legs generally surrounding the insect pin. Other specimens were preserved in 96% ethanol (ETOH). Some specimens were slide mounted in Euparal; the genitalia of males and ovipositors of females were cleared overnight in approximately 10% potassium hydroxide (KOH) and preserved in microvials filled with glycerol on the same pin as the dry insect, or on a separate pin if the crane fly was preserved in ETOH.

**Table 1.** Collecting sites in Korea.

Locality	Year	Coordinates (N*, E*)	Collector	Method	Collection
S. Korea, Suigen, Chosen	1930	37°16.00'N, 127°01.00'E	I. Tabashi	Net	USNM
N. Korea, Ompo (Onbo, Hamgyeongbuk-do,Gyeongsung-gun)	1937 1938 1939	41°30.81'N, 129°34.69'E	A. M. Yankovsky	Net	USNM
N. Korea, Seren Mts. (Hamgyeongbuk-do, Gyeongsung-gun)	1938	41°41.24'N, 129°18.55'E	A. M. Yankovsky	Net	USNM
N. Korea, Kankyo Nando, Puksu Pyaksan (Yanggang-do, Pungseo-gun, Mt. Buksubaeksan)	1939	40°41.99'N, 127°42.96'E	A. M. Yankovsky	Net	USNM
N. Korea, Chonsani (Yanggang-do, Daehongdan-gun)	1940	41°59.62'N, 128°45.15'E	A. M. Yankovsky	Net	USNM
S. Korea, #12, Hwy. #20, 8 mi. SW of Kangnung (Gangwon-do, Gangneung, Seongsan-myeon, Eoheul-ri)	1954	37°42.00'N, 128°47.00'E	G. W. Byers	Net	USNM, SMEK
S. Korea, #25, #26 Central National Forest, 18 mi. NE Seoul (Gyeonggi-do, Namyangju-ai, Sudong-myeon, Naebang-ri)	1954	37°44.89'N, 127°17.62'E	G. W. Byers	Net	SMEK, USNM
N. Korea, Prov. South Pyongan, Pyongyan, Hotel garden	1971	39°00.63'N, 125°45.10'E	S. Horvatovich, J. Papp	Net	HNHM
S. Korea, Gyeonggi-do, Paju-si, Aengmubong	1973	37°45.46'N, 126°55.65'E	Y. Kim	Net	KU
S. Korea, Gyeonggi-do, Pocheon-si, Soheul-eup, Gwangneung Forest	1973	37°45.05'N, 127°09.70'E	O. Lee	Net	KU
S. Korea, Seoul, Mt. Suraksan	1974	37°41.79'N, 127°04.93'E	–	–	KU
S. Korea, Gyeongsangbuk-do, Yeongju-si, Punggi-eup, Samga-ri, Mt. Sobaeksan	2000 2001	36°55.28'N, 128°30.33'E	–	–	KU
S. Korea, Gyeongsangbuk-do, Bonghwa-gun, Mt. Seondalsan	2000 2001	37°02.38'N, 128°42.55'E	–	–	KU
S. Korea, Gyeongsangbuk-do, Yeongyang-gun, Ilwol-myeon, Yonghwa-ri, Mt. Ilwolsan, Yonghwasa Temple	2001	36°48.71'N, 129°07.55'E	–	–	KU
S. Korea, Jeollabuk-do, Namwon, Sannae-myeon, Buun-ri, Namwonsi Sannaemyeon Baemsagol	2009	35°21.21'N, 127°34.95'E	S. W. Jung	Net	KU
S. Korea, Gangwon-do, Jeongseon-gun, Imgye-myeon, Dojeon-ri	2011	37°32.15'N, 128°54.17'E	H.-W. Byun et al.	Malaise trap	NIBR
S. Korea, Gangwon-do, Pyeonchang-gun, Jinbu-myeon, Dongsan-ri, Odaesan NP	2012	37°44.26'N, 128°35.50'E	S. Podenas	Net	NIBR
S. Korea, Jeollanam-do, Gurve, Masan-myeon, Hwangjeon-ri	2013	35°14.62'N, 127°29.38'E	S. Podenas, H.-W. Byun	Net	NIBR
S. Korea, Gangwon-do, Chuncheon, Dongsan-myeon, Bongmyeong-ri, KNU experimental Forest	2014	37°46.74'N, 127°48.94'E	S. Podenas	Net	NIBR
S. Korea, Gyeonggi-do, Gapyeong-gun, Buk-myeon, Jeokmok-ri	2014	37°58.61'N, 127°26.59'E	D.-G. Kim, M.-D. Baek, H.-D. Gang, Ch. Uy	Net	KU
S. Korea, Gyeonggi-do, Gapyeong-gun, Buk-myeon, Jeokmok-ri, Garim-gyo (Br.)	2015	37°58.55'N, 127°26.49'E	Y. J. Bae	Malaise trap	KU
S. Korea, Jeollanam-do, Gurye-gun, Toji-myeon, Naeseo-ri, Piagol valley	2015	35°16.31'N, 127°34.29'E	S. Podenas	Net	NIBR
	2016	35°16.40'N, 127°34.15'E			
	2019	35°15.50'N, 127°34.93'E			
		35°15.95'N, 127°34.85'E			
		35°16.03'N, 127°34.66'E			
S. Korea, Gyeongsangbuk-do, Gyeongju-si, Yangbuk-myeon, Janghang-ri	2016	35°45.74'N, 129°21.84'E	S. Podenas, H. M. Baek	Net	NIBR
S. Korea, Gyeonggi-do, Yangpyeong, Cheongun-myeon, Dowon-ri	2017	37°32.70'N, 127°47.69'E	S. Podenas	At light	NIBR
S. Korea, Gyeonggi-do, Paju-si, Gunnae-myeon, Jeongja-ri, Warrior Base Training Area	2017	37°55.07'N, 126°44.50'E	T.A. Klein, H.-C. Kim	NJ trap	NIBR

Information on the examined material is given as it is on the labels, except coordinates, altitudes and measurement units which are given according to journal requirements. Also given are any additional labels kept with the specimen or additional notes on the same label, such as “metatype” written by Dr. Ch. P. Alexander, who originally described the species. For specimens collected by S. Podenas and his colleagues, the collecting date on the label is followed by a number in brackets. Different localities where insects were collected on the same date were given separate numbers and all information from those localities, whether in the field notes, databases, photographs, or other locality information, were marked with the specific number. Specimens are arranged according to the collecting date.

Prior to these studies all East Palaearctic and most Oriental species of Hexatoma (Eriocera) were studied and photographed. Special attention was payed to species recorded from neighbouring countries, like China, Japan, and Russia. Only four East Palaearctic species were not accessible to the authors of this publication and other entomologists who kindly helped with illustrations or photographs. These species are H. (E.) caesia (Savchenko, 1979), H. (E.) cleopatroides (Men, 2015) (Men and Yu 2015), H. (E.) flavimarginata (Yang, 1999), and H. (E.) superba (Savchenko, 1976) (Savchenko and Krivolutskaya 1976), but Chinese species are well illustrated and included in the key that covers all Chinese species (Men and Yu 2015), and Russian species also are included in the key and described in detail (Savchenko and Krivolutskaya 1976).

Crane flies were observed using an Olympus SZX10 dissecting microscope. Photographs were taken with a Canon EOS R5 digital camera through a Canon MP–E 65 mm macro lens and through Mitutoyo M Plan Apo 10× and 20× lenses mounted on the same camera.

The terminology of adult morphological features generally follows that of Cumming and Wood (2017), de Jong (2017) for terminology of wing venation.

The general distribution of species is given according to Oosterbroek (2022).

## ﻿Taxonomy

### ﻿ Hexatoma

Taxon classificationAnimaliaDipteraLimoniidae

Latreille, 1809

7E35892C-1FB7-5D01-97B6-BDCFFA69AC9C

Hexatoma Latreille 1809: 260; Edwards 1938: 63 (in key), 64 (descriptive note), pl. 3, fig. 14; Alexander 1948: 528 (in catalogue); Ishida 1959: 2 (in key); Savchenko and Krivolutskaya 1976: 76 (note on distribution); Savchenko 1983: 67 (note on distribution); Savchenko 1986: 337–342 (redescription), figs 96, 173–179; Savchenko 1989: 118–119 (redescription), figs 58–60.
Nematocera Meigen 1818: 209, pl. 7, figs 1–4.
Anisomera Meigen 1818: 210, pl. 7, figs 5–8.
Peronecera Curtis 1836: 589, figs 2–7; Enderlein 1936: 22 (in key), fig. 41.
Trimacromera Enderlein 1936: 23 (in key), fig. 43.

#### Type species.

*Hexatomanigra* Latreille, 1809 (southern Europe).

#### Description.

Medium-sized to large crane flies with body length 6.5–32.0 mm and wing length 7.5–21.0 mm. Body coloration varies from yellow or orange to brown and black, some species have very distinct coloration.

***Head*.** Rounded posteriorly without neck–like extension. Vertex wide with distinct tubercle. Length of antenna varies from short, hardly reaching wing base, if bent backwards, to very long, when it exceeds body length up to 4×. Antennae sexually dimorphic. Males usually have longer antennae than females, but that is because of elongated basal segments of the male flagellum. Antenna has reduced number of segments, less than typical 14–16-segmented antenna of most short–palped crane flies, often male antenna 6- or 7-segmented, that of female 8–11-segmented. Verticils missing or indistinct, but male flagellum often with two longitudinal rows of short erect spines medially.

***Thorax*.** Some species with very setose thorax, setae could be long, dense, and erect. Some species with more dense and longer pubescence in males than in females. Prothorax very narrow but wide. Mesonotal prescutum usually without, sometimes with, small indistinct tubercular pits, pseudosutural fovea small. Prescutum and presutural scutum with three or four longitudinal stripes. Pleuron usually without stripes, could be bare or setose, depending on species. Meron usually big, thus middle and posterior coxae widely separated. Wing long and narrow, patternless or with very distinct pattern, sometimes completely dark, even black, but often with light “window” in the middle, stigma present or missing. Macrotrichiae missing on wing cells. Arculus present, humeral vein close to arculus. Vein Sc long, reaching wing margin far beyond branching point of Rs, sc-r slightly before tip of Sc. Radial sector with two or three branches reaching wing margin. R_1_ short, nearly transverse, or slightly elongate, R_3_ and R_4_ diverging. Cell r_3_ with long stem. Cell m_1_ present or missing; two, three or four branches of M reaching wing margin. Discal cell present or missing. Position of cross-vein m-cu differs according to species. Vein CuP usually slightly arched at distal part, anal vein long, slightly sinuous or arched, reaching wing margin close to the level of Rs base. Anal angle distinct, widely rounded. Wing cells without macrotrichiae. Wing squama setoseless. All legs with tibial spurs, usually fore leg with single spur, middle and posterior legs with two spurs each. Claw simple or with single subbasal spine.

***Abdomen*.** Tergites with paired transverse sutures. Male terminalia approximately as wide as the rest of the abdominal segments, slightly elongate. Epandrium (ninth tergite) wider than longer, posterior margin simple without additional structures. Each gonocoxite elongate, two pairs of terminal gonostyli, the shape of which are only slightly variable among different species. Aedeagus simple, short, and straight. Ovipositor usually with long and narrow cerci and hypovalvae, distal part of cercus slightly raised upwards, acute. Some species with shortened ovipositor bearing fleshy cerci and hypovalvae.

596 species belong to the genus *Hexatoma* worldwide, they are divided into six subgenera:

H. (Eriocera) Macquart, 1838 (556 extant and three fossil species),

H. (Cladolipes) Loew, 1865 (three species, one of them with two subspecies),

H. (Coreozelia) Enderlein, 1936 (one Western Palearctic species),

H. (Euhexatoma) Alexander, 1936 (one Oriental species),

H. (Hexatoma) Latreille, 1809 (23 species, one of them with two subspecies),

H. (Parahexatoma) Alexander, 1951 (12 species, Afrotropics only) (Oosterbroek 2022).

Six fossil species are described from the Eocene, three of them in H. (Eriocera), three not assigned to subgenera (Evenhuis 2014).

### ﻿Key to subgenera of the genus *Hexatoma* Latreille

**Table:** 

1	Radial sector with three branches (Figs 2–6, 16, 24, 25, 29, 35, 43, 48, 51, 56)	**2**
–	Radial sector with two branches (Fig. 1)	**Hexatoma (Cladolipes) Loew, 1865**
2	Discal cell present (Figs 2–4, 16, 24, 29, 35, 43, 48, 51, 56), missing in exceptionally rare cases in atypical specimens (Fig. 25), 3 (Figs 2, 3, 24, 25, 29, 48, 51) or 4 (Figs 4, 16, 35, 43, 56) branches of M reaching wing margin	**3**
–	Discal cell missing, two branches of M reaching wing margin (Figs 5, 6)	**5**
3	Supernumerary cross-veins missing in cells r_3_, r_4_ and r_5_ (Figs 2, 3, 16, 24, 25, 29, 35, 43, 48, 51, 56)	**4**
–	Supernumerary cross-veins in cells r_3_, r_4_ and r_5_ (Fig. 4)	**Hexatoma (Euhexatoma) Alexander, 1936**
4	Vein Sc reaching wing margin beyond Rs branching point, R_2_ beyond fork of R_3_ and R_4_ (Figs 3, 16, 24, 25, 29, 35, 43, 48, 51, 56)	**Hexatoma (Eriocera) Macquart, 1838**
–	Vein Sc reaching wing margin at Rs branching point, R_2_ at fork of R_3_ and R_4_ (Fig. 2)	**Hexatoma (Coreozelia) Enderlein, 1936**
5	Ovipositor short with fleshy valves	**Hexatoma (Hexatoma) Latreille, 1809**
–	Ovipositor with long and slender valves	**Hexatoma (Parahexatoma) Alexander, 1951**

### ﻿ Hexatoma (Eriocera)

Taxon classificationAnimaliaDipteraLimoniidae

Macquart, 1838

04335E21-01F5-5019-BE29-15B185118096

**Figures 1–6.**
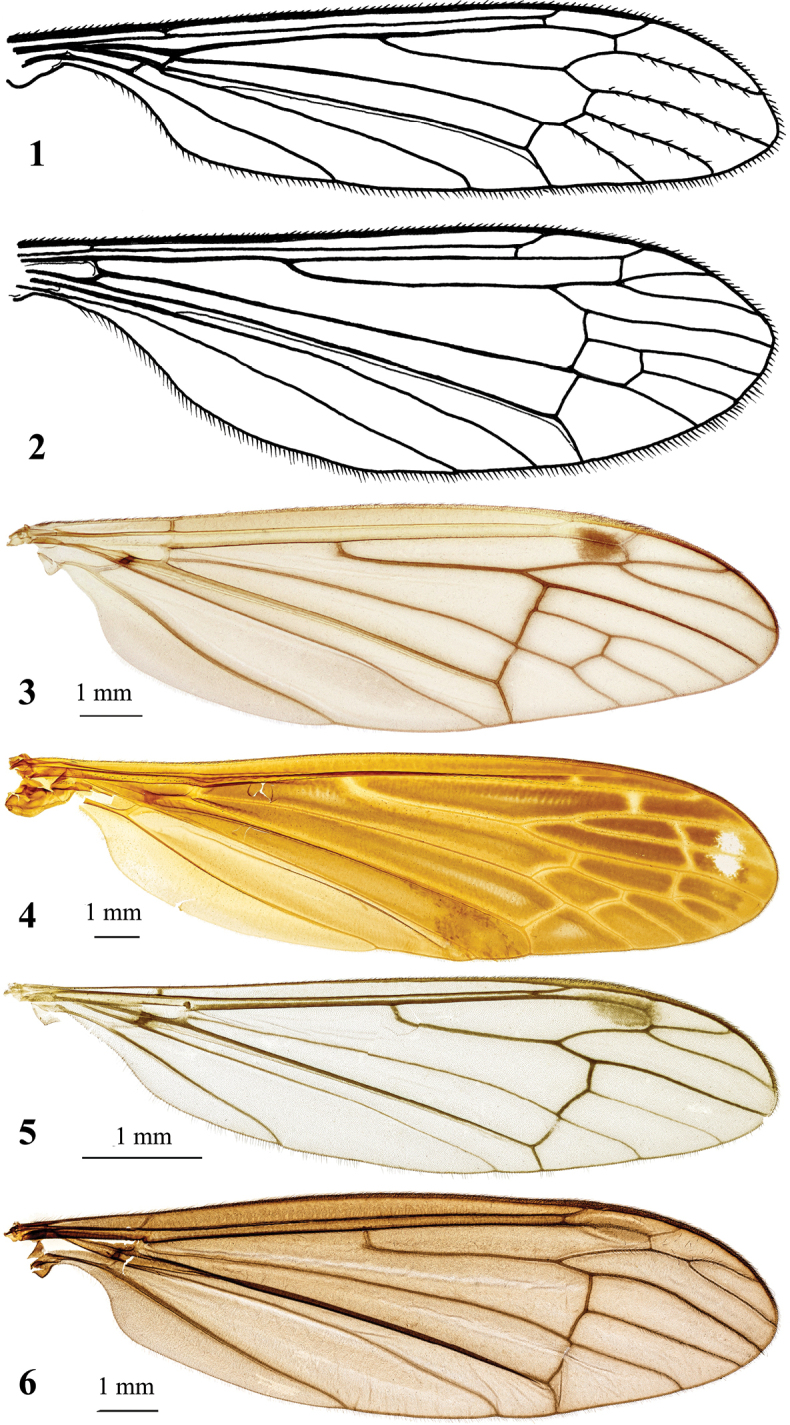
*Hexatoma* wings **1**H. (Cladolipes) simplex (Loew, 1865) **2**H. (Coreozelia) cimicoides (Scopoli, 1763) **3**H. (Eriocera) gifuensis Alexander, 1933 **4**H. (Euhexatoma) triphragma Alexander, 1936, holotype **5**H. (Hexatoma) khasiensis Alexander, 1962, holotype **6**H. (Parahexatoma) angustatra Alexander, 1963, holotype. Scale bars: 1.0 mm. (**1** redrawn after Mendl (1979) and Savchenko (1989); **2** redrawn after Slipka (1949)).

Hexatoma (Eriocera) Macquart, 1838: 78, pl. 10, fig. 2; Alexander 1948: 528–529 (in catalogue); Ishida 1959: 2 (in key); Savchenko 1983: 67 (note on distribution); 1986: 342–344 (descriptive note), figs 174,1–2, 176,1–3, 178,1–2; 1989: 121 (descriptive note), figs 58,1, 59,1–2, 60,1.
Caloptera Guerin–Meneville 1831: 20 (nom. obl.).
Eriocera Macquart 1838: 78; Edwards 1921: 67–70 (redescription), pl. 10, figs 1–12.
Evanioptera Guerin–Meneville 1838: 287.
Pterocosmus Walker 1848: 78.
Allarithmia Loew 1850: 36, 38.
Oligomera Doleschall 1857: 387.
Physecrania Bigot 1859: 123.
Arrhenica Osten Sacken 1860: 243–244.
Penthoptera Schiner 1863: 220.
Androclosma Enderlein 1912: 34–35, fig. U.
Globericera Matsumura 1916: 471.
Coreozelia Enderlein 1936: 22 (in key), fig. 40.

#### Type species.

*Hexatomamacquarti* (Enderlein, 1912) (= *Erioceranigra* Macquart, 1838, = *Hexatomamacquarti* (Enderlein, 1912)) (Brazil).

#### Description.

Most characters as for the genus. Medium-sized to large crane flies with body length 6.5–32.0 mm and wing length 7.5–21.0 mm. Most species dark colored, but some could be orange-yellow (e.g., *H.masakii* Alexander, 1934).

***Head*.** Rounded, vertex with distinct tubercle. Antennae sexually dimorphic. Male antenna longer than that of female, sometimes few times longer than body, 6- or 7-segmented, female antenna 8–11-segmented. Verticils missing or indistinct, but male flagellum often with two longitudinal rows of short erect spines.

***Wing*.** Radial sector with three branches, discal cell always present, three or four branches of M reaching wing margin.

**Figures 7–11.**
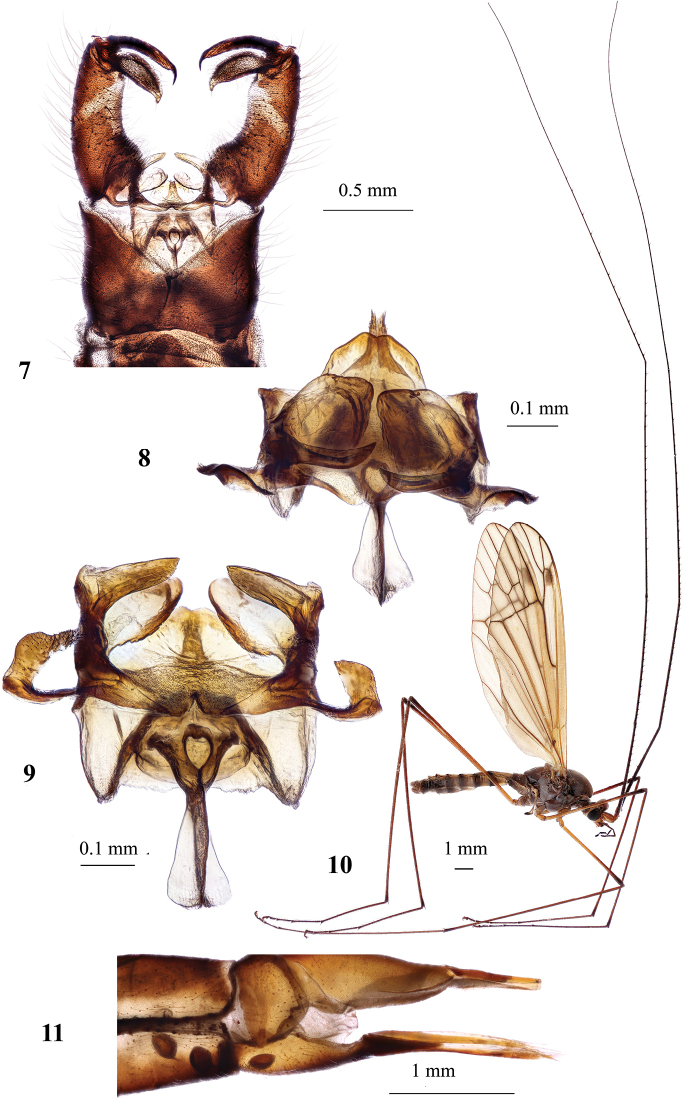
Hexatoma (Eriocera) gifuensis Alexander, 1933 **7** male genitalia, dorsal view **8** aedeagal complex, fronto–dorsal view **9** aedeagal complex, dorsal view **10** male, general vew **11** ovipositor, lateral view (tip of cercus broken). Scale bars: 0.5 mm (**7**); 0.1 mm (**8, 9**); 1.0 mm (**10, 11**).

***Terminalia*.** Male terminalia slightly elongate, not wider than preceding abdominal segments. Epandrium transverse, posterior margin slightly concave. Gonocoxite elongate with two pairs of terminal gonostyli. Outer gonostylus long, narrow with spine-shaped apex. Inner gonostylus long, fleshy, and setose. Aedeagus simple, usually short, and straight (Figs 7–9, 17, 18, 22, 23, 30, 31, 40, 44, 52, 53), but could be long (Figs 36, 37) and arched (Figs 57, 58). Paramere usually two–branched and variable among species. Ovipositor with long and narrow cercus and long hypovalva, distal part of cercus slightly raised upwards, acute, some species with subapically dilated hypovalva.

Subgenus H. (Eriocera) includes 556 extant species (seven of them with two subspecies each). It has a worldwide distribution with the highest diversity in the Oriental region, 286 species (four of them with two subspecies each), the Neotropics, 143 species, and the Eastern Palearctic, 65 species. Thirty-three species (one of them with two subspecies) are recorded from Nearctic, 29 species (one with two subspecies) from Afrotropics, five species from Australasia, and four species from West Palaearctic (Oosterbroek 2022). Three fossil species are described from Eocenian Baltic amber (Evenhuis 2014).

##### List of Korean *Hexatoma* crane flies

Hexatoma (Eriocera) gifuensis Alexander, 1933

Hexatoma (Eriocera) ilwola Podenas, sp. nov.

Hexatoma (Eriocera) masakii Alexander, 1934

Hexatoma (Eriocera) pernigrina Alexander, 1938

Hexatoma (Eriocera) pianigra Podenas, sp. nov.

Hexatoma (Eriocera) serenensis Podenas, sp. nov.

Hexatoma (Eriocera) stackelbergi Alexander, 1933

Hexatoma (Eriocera) ussuriensis Alexander, 1934

### ﻿Key to Korean species of the genus *Hexatoma* Latreille

**Table:** 

1	Entire body, including legs and wings, coal black (Figs 26, 33)	**2**
–	Body patterned with brown, gray, or orange (Figs 10, 12, 21, 41, 45, 47, 50)	**3**
2	Wing cell m_1_ missing (Fig. 29). Gonocoxite short and wide, length just slightly exceeds width (Figs 30, 31). Cercus of ovipositor comparatively short, triangular (Fig. 32). Wing length up to 15.0 mm	**Hexatoma (Eriocera) pernigrina Alexander, 1938**
–	Wing cell m_1_ present (Fig. 35). Gonocoxite long and narrow, length few times exceeds width (Figs 36, 37). Cercus of ovipositor long, parallel-sided (Fig. 38). Wing length above 16.0 mm	**Hexatoma (Eriocera) pianigra Podenas, sp. nov.**
3	Male	**4**
–	Female	**9**
4	Wing cell m_1_ present (Figs 16, 43). Wing length above 16.0 mm	**5**
–	Wing cell m_1_ missing (Figs 3, 24, 25, 48, 51). Wing length up to 13.0 mm	**6**
5	Antenna 3× as long as the rest of the body (Fig. 41). Cell m_1_ distinctly longer than its stem (Fig. 43)	**Hexatoma (Eriocera) serenensis Podenas, sp. nov.**
–	Antenna distinctly shorter than the rest of the body (Fig. 12). Cell m_1_ approximately as long as its stem (Fig. 16)	**Hexatoma (Eriocera) ilwola Podenas, sp. nov.**
6	Antenna at least twice as long as entire body, usually more than that (Figs 10, 21, 50)	**7**
–	Antenna not reaching wing root if bent backwards	**Hexatoma (Eriocera) stackelbergi Alexander, 1933**
7	Abdomen orange yellow (Fig. 21). Costal wing area darkened (Figs 24, 25). Outer gonostylus with hook-shaped apex (Figs 22, 23)	**Hexatoma (Eriocera) masakii Alexander, 1934**
–	Abdomen brown or dark brown (Figs 10, 50). Costal wing area not darker than the rest of the wing (Figs 3, 51)	**8**
8	Paramere with dorsal branch parallel-sided, lower branch wide, plate-shaped, anterior apodeme of aedeagus with wide lateral lobes (Figs 8, 9)	**Hexatoma (Eriocera) gifuensis Alexander, 1933**
–	Paramere with dorsal branch wedge-shaped, lower branch elongate, anterior apodeme of aedeagus without lateral plates (Figs 52, 53)	**Hexatoma (Eriocera) ussuriensis Alexander, 1934**
9	Abdomen orange yellow. Costal wing area darkened (Figs 24, 25)	**Hexatoma (Eriocera) masakii Alexander, 1934**
–	Abdomen brown or dark brown (Figs 45, 47). Costal wing area not darker than the rest of the wing (Figs 3, 16, 43, 48, 51)	**10**
10	Wing cell m_1_ missing (Figs 3, 48, 51)	**11**
–	Wing cell m_1_ present (Figs 16, 43)	**13**
11	Wing stigma distinct, dark brown (Figs 3, 51)	**12**
–	Wing stigma very small, nearly missing (Fig. 48)	**Hexatoma (Eriocera) stackelbergi Alexander, 1933**
12	Wing stigma elongate, oval, radial sector arched at base (Fig. 51)	**Hexatoma (Eriocera) ussuriensis Alexander, 1934**
–	Wing stigma approximately as long as wide, radial sector angulate at base (Fig. 3)	**Hexatoma (Eriocera) gifuensis Alexander, 1933**
13	Thorax brown (Fig. 13). Cell m_1_ approximately as long as its stem (Fig. 16)	**Hexatoma (Eriocera) ilwola Podenas, sp. nov.**
–	Thorax gray (Fig. 42). Cell m_1_ distinctly longer than its stem (Fig. 43)	**Hexatoma (Eriocera) serenensis Podenas, sp. nov.**

### ﻿ Hexatoma (Eriocera) gifuensis

Taxon classificationAnimaliaDipteraLimoniidae

Alexander, 1933

19AAA013-C8F3-56A5-9C3B-5CA5C7FAA7B5

Figs 3 , 7–11 , 59

Hexatoma (Eriocera) gifuensis Alexander 1933: 153–155, pl. 1, figs 15–16, pl. 2, fig. 33.

#### Type material examined.

***Holotype***, male (wing and genitalia slide mounted), **Japan**, Gifu, 6 June 1931, Kariya leg. (USNM). ***Allotype***, female (antenna, wing and ovipositor slide mounted on same slide as holotype), topotypic (USNM).

#### Other examined material

(Fig. 59). **South Korea**, 1 female (in ETOH), Gyeonggi–do, Paju–si, Gunnae–myeon, Jeongja–ri, Warrior Base Training Area, 37°55.07'N, 126°44.50'E, alt. 20 m, 18 July 2017, T. A. Klein, H.–C. Kim leg., NJ trap (NIBR); 1 female, 1 specimen, sex unknown (in ETOH), same collection data as for preceding, 25 July 2017 (NIBR); **Japan**, 1 male (marked as metatype; antenna, leg and wing slide mounted), Shikoku, Matsuyama, Iyo, 14 September 1947, T. Ishihara leg. (USNM); 1 male (pinned, genitalia in microvial with glycerol), Niigata, 16 August 2021, coll. D. Kato (NRC).

#### Description.

***Body*** dark brown. Male body length 9.5 mm, wing length 14.3 mm. Female body length 12.5–13.5 mm, wing length 12.0–12.2 mm.

***Head*.** Dark brown, postero–laterally yellowish. Vertical tubercle large, dark brown, yellowish laterally. Eyes widely separated, distance between them at the base of the antennae nearly the same as length of both basal antennomeres. Male antenna 47.3 mm long, ~ 3× as long as the entire body (Fig. 10). Antennal scape elongate, nearly cylindrical, brownish yellow with short and erect dark brown setae dorsally and ~ 3× as long as pedicel. Pedicel small, subglobular, brownish yellow. Rostrum brown. Palpus and mouth parts dark brown.

***Thorax*.** Cervical sclerites dark brown. Pronotum short but wide, brown. Prescutum and presutural scutum grayish brown with three longitudinal dark brown stripes. Medial stripe separated anteriorly by narrow grayish line, which is missing posteriorly. Tubercular pits missing, pseudosutural fovea brown. Dorsopleural membrane dark brown, yellowish anteriorly. Postsutural scutum with each lobe brown with concave elongate dark brown spot in middle, area between lobes brown. Scutellum dark brown, lighter along posterior margin. Mediotergite entirely brown. Pleuron uniformly dark brown. Episternum bare, setoseless. Meron comparatively small, second and third pairs of legs close together. Wing (Fig. 3) with brownish tinge, costal area darker, all veins narrowly surrounded with darker brownish. Stigma distinct, dark brown, short, just slightly longer than wide. Veins brown, yellow in costal area. Venation: Sc long, reaching wing margin slightly beyond r-m, sc-r approximately at r-m. Radial sector long, nearly straight, slightly arched or angulate at base, if angulate then with very short spur. Free end of R_1_ concave, R_2_ close to R_1_ apex. R_3_ and R_4_ diverging towards wing margin, cell r_3_ with long stem, which is half as long as Rs. Cross-vein r-m distinct, transverse, in alignment with basal deflection of M_1_ (base of discal cell). Discal cell 1.8× longer than wide. Cross-vein m-cu slightly beyond base of discal cell. Vein CuP distinctly curved at distal part, thus cell cup gets wider towards wing margin. Anal vein long, slightly concave in middle, apex reaching wing margin at the level of Rs base. Anal angle wide, posterior margin widely rounded. Halter pale with black knob and slightly darkened base of stem. Length of male halter 1.5 mm, that of female 1.3 mm. Coxae dark brown dorsally, yellowish ventrally and posteriorly. Trochanters obscure yellow. Femur yellow with narrowly blackened distal part. Tibia brownish yellow with narrowly darkened apex. Basal tarsomere brownish with yellow base, remainder of tarsus brown to dark brown or black. Covered with long dense dark brown setae. Male femur I: 5.5 mm long, II: 4.3 mm, III: 7.9 mm, tibia I: 7.9 mm, II: 9.9 mm, III: 11.3 mm, tarsus I: 7.4 mm, II: 10.3 mm, III: 7.2 mm. Tibia of fore leg with single apical spur, tibiae of middle and hind pairs of legs with two apical spurs each.

***Abdomen***. Tergites dark brown, narrowly yellow laterally, with two pairs of transverse indistinct sutures. Sternites dark brown along middle, yellow laterally. Male terminalia (Fig. 7) dark brown to black. Epandrium wider than long, posterior margin with deep and wide V-shaped emargination. Gonocoxite elongate, 2× longer than wide at base, dorsal surface with narrow pale stripe across middle. Two pairs of long narrow gonostyli. Outer gonostylus sclerotized, long, slightly arched, apex spine-shaped. Inner gonostylus elongate, fleshy and setose, spindle shaped. Paramere bilobed, outer lobe elongate, knife-shaped, distal part slightly arched, lower lobe wide, plate-shaped (Figs 8, 9). Aedeagus simple, short and straight, bifid at apex. Aedeagal sheath completely covers aedeagus dorsally. Anterior apodeme long with membranous lobes along both sides, extending far beyond frontal margin of aedeagal sheath. Ovipositor (Fig. 11) brownish yellow, valvae long and narrow.

#### Elevation range in Korea.

Ca. 20 m altitude.

#### Period of activity in Korea.

Second half of July.

#### Habitat.

Unknown. Attracted to light.

#### General distribution.

Honshu and Shikoku islands of Japan. Recorded in the Korean Peninsula for the first time.

### ﻿ Hexatoma (Eriocera) ilwola

Taxon classificationAnimaliaDipteraLimoniidae

Podenas sp. nov.

FAAA46CD-1C60-57BA-AEAA-764D195F9601

http://zoobank.org/B06AD1FA-08E1-4074-AA77-2AF768A9EA48

Figs 12–19 , 60

#### Type material examined

(Fig. 60). ***Holotype***, male (in ETOH), **South Korea**, Gyeongsangbuk–do, Yeongyang–gun, Ilwol–myeon, Yonghwa–ri, Mt. Ilwolsan, Yonghwasa Temple, 36°48.71'N, 129°07.55'E, alt. 510 m, 6 August 2001 (KU). ***Paratypes***: **North Korea**, 1 female (pinned), Ompo, alt. 230 m, 28 August 1939, A. Yankovsky leg. (USNM); **South Korea**, 1 female (in ETOH), topotypic (KU); 2 females (in ETOH), Gyeongsangbuk–do, Yeongju–si, Punggi–eup, Samga–ri, Mt. Sobaeksan, 36°55.28'N, 128°30.33'E, alt. 400 m, 13 August 2001 (KU); 1 female (pinned), Gangwon–do, Chuncheon–si, Dongsan–myeon, Bongmyeong–ri, KNU experimental forest, 37°46.74'N, 127°48.94'E, alt. 230 m, 22 August 2014 (1), S. Podenas leg. (NIBR).

#### Diagnosis.

Large brownish gray species with body length 19.0–31.8 mm. Rostrum brown. Head and thorax with short and scarce pubescence. Male antenna reaching to approximately middle of abdomen if bent backwards. Prescutum and presutural scutum with four distinct dark brown stripes. Wing translucent with distinct stigma. Cell m1 present. Halter with dark knob. Femur yellow with narrowly blackened distal part. Abdominal sternites yellowish. Epandrium of male genitalia with wide V-shaped emargination. Gonostyli approximately equal in length. Posterior margin of inner gonostylus rounded, apical part slightly arched. Paramere V-shaped. Aedeagus simple, short, straight. Ovipositor with nearly straight cercus. Hypovalva long, distal part widened and setose, apex distinctly narrows into setiforme structure.

#### Etymology.

Species is named after type locality, Ilwol mountain.

#### Description.

Body coloration brownish gray. Body length of male 19.0 mm, female 23.0–31.8 mm, wing length of male 20.8 mm, female 16.3–20.6 mm.

***Head*** (Fig. 13). Dark brown, dusted with gray, pale gray along eye margin, densely covered with short erect brown setae dorsally. Vertical tubercle large, rounded, with indistinct median vita, reddish brown fronto-laterally above base of antenna. Eyes widely separated in both sexes, distance between them at base of antennae equals to length of scape and pedicel taken together. Male antenna (Fig. 14) entirely brown, 7-segmented, 12.4 mm long, reaching to approximately middle of abdomen if bent backwards. Scape large, twice as long as wide, sparsely dusted with gray. Pedicel subglobular. Flagellomeres with two parallel lines of short spines medially. Basal flagellomere approximately as long as head and both basal antennomeres taken together, remaining flagellomeres getting longer towards apex of antenna. Female antenna (Fig. 15) 11-segmented, 4.8–6.5 mm long, reaching wing base if bent backwards. Scape elongate, cylindrical, 1.6× longer than wide and 3× as long as pedicel. Pedicel wider than long. Basal flagellomere 1.75× as long as scape, remaining flagellomeres decreasing in length, apical segment elongate, approximately as long as preceding segment. Comparative length of flagellomeres slightly varies depending on specimen. Short spines that are present on male flagellum are completely missing on female antenna. Rostrum, palpus and mouth parts brown, just distal palpomeres somewhat darker.

***Thorax*.** Cervical sclerites brown, dusted with gray. Pronotum much wider than long, gray with narrowly yellowish anterior margin. Prescutum light bluish gray, presutural scutum bluish gray laterally, brownish gray posteriorly. Prescutum and presutural scutum with four distinct dark brown stripes (Fig. 13) and covered with comparatively sparse medium long erect yellowish setae, that are less dense and shorter than in *H.aequinigra*, but denser and longer than in *H.superba*. Area separating medial stripes approximately as wide as stripe itself. Tubercular pits small, close to each other at anterior part of sclerite, pseudosutural fovea small, brownish. Postsutural scutum with each lobe dark brown with gray margins. Area between lobes brown. Scutellum dark brown, dusted with gray, posterior and lateral margins gray and covered with long yellow setae. Mediotergite gray because of dense pruinosity, dark brown posteriorly. Pleuron brown dorsally, whitish gray ventrally, covered with fine yellowish setae. Wing (Fig. 16) slightly iridescent, with brownish tinge, yellowish in costal area and at base. No other dark spots except elongate stigma. Veins light brown. Macrotrichiae on distal veins very scarce, nearly missing. Venation: humeral vein slightly before arculus, Sc very long, reaching wing margin distinctly beyond branching point of R_2+3_ and R_4_, sc-r close to the apex of Sc. Rs long and nearly straight, arched at base. Free end of R_1_ elongate, R_2_ twice its own length before apex of R_1_. R_3_ and R_4_ diverging, cell r_3_ with stem, which is nearly as long as m-cu. Cross-vein r-m distinct, transverse, slightly beyond base of discal cell. Discal cell 2× longer than wide. Cross-vein m-cu at middle of discal cell length. Anal vein long, slightly concave in the middle, apex distinctly beyond the level of Rs base. Anal angle wide, posterior margin widely rounded. Stem of halter grayish brown with yellowish base, knob dark brown. Length of male halter 2.4 mm, that of female 1.9–2.3 mm. Coxae brownish yellow, densely covered with whitish–bluish gray pruinosity and long yellowish setae. Posterior coxa somewhat darker. Trochanters obscure yellow. Femur yellow with narrowly blackened distal part. Tibia brownish yellow with slightly darkened apex. Tibia of fore leg with single apical spur, tibiae of middle and hind pairs of legs with two apical spurs each. Basal tarsomere light brown with darker distal part, remainder of tarsus brown to dark brown. Male femur I: 9.5 mm long, II: 11.5 mm, III: 15.7 mm, tibia I: 14.0 mm, II: 9.5 mm, III: 17.0 mm, tarsus I: 16.2 mm, II: 12.7 mm, III: 12.0 mm. Female femur I: 9.0–10.5 mm long, II: 10.0–11.5 mm, III: 14.0–14.2 mm, tibia I: 11.0–12.2 mm, II: 10.0–10.4 mm, III: 13.0–13.5 mm, tarsus I: 11.0–12.0 mm, II: 8.5–10.2 mm, III: 7.8–8.5 mm long. Claw dark brown basally, reddish brown distally, simple, without spines.

***Abdomen*.** Tergites dark brown, dusted with gray, narrowly orange along lateral margin, posterior margin narrowly orange starting from fourth tergite. All tergites with two pairs of transverse sutures and covered with very short yellowish setae. Sternites dark brown basally, obscure yellow laterally and posteriorly, dusted with gray. Male terminalia (Fig. 17) brownish yellow, slightly narrower than pregenital segments. Epandrium wider than long, posterior margin with wide V-shaped emargination. Gonocoxite elongate, slightly more than twice as long as wide at base, dorsal surface uniformly sclerotized. Two pairs of long narrow gonostyli. Outer gonostylus sclerotized, long, slightly arched, apex distinctly narrowed and spine-shaped. Inner gonostylus elongate, fleshy and setose, posterior margin rounded, apical part slightly arched. Paramere (Fig. 18) bifid, V-shaped, dorsal branch wider at base, tip folded, ventral branch straight and narrow. Aedeagus simple, short, and straight, protruding through aedeagal sheath in dorsal view, apex bifid. Anterior apodeme long and narrow, extending forward beyond frontal margin of aedeagal sheath. Female pregenital segment and ovipositor orange (Fig. 19). Tenth tergite elongate. Cercus slightly darker at base, nearly straight, rounded apex, distal part slightly raised upwards, very apex pale. Hypovalva long, parallel-sided at ~ 2/3 from base, distal part widened and setose, reaching to ~ 1/3 of cercus, apex distinctly narrows into setiforme structure.

**Figures 12–19.**
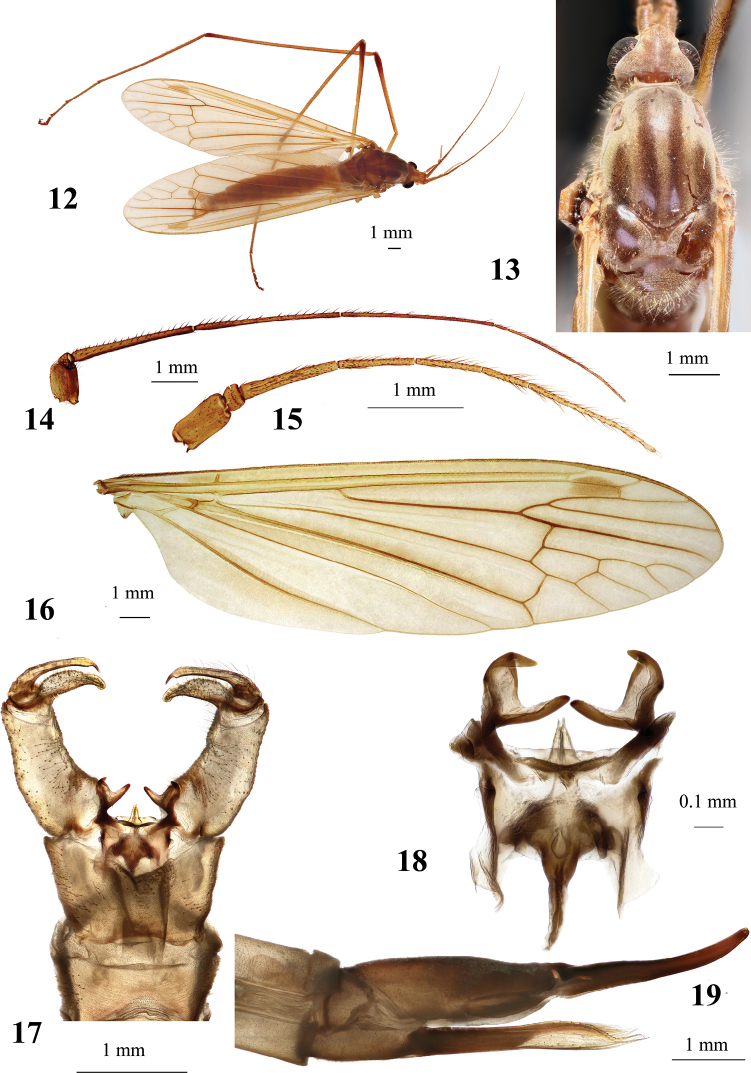
Hexatoma (Eriocera) ilwola Podenas, sp. nov. **12** holotype, male, dorsal view **13** head and thorax, dorsal view, paratype, female **14** male antenna, holotype **15** female antenna, paratype **16** female wing, paratype **17** male genitalia, dorsal view, holotype **18** aedeagal complex, dorsal view **19** ovipositor, lateral view, paratype. Scale bars: 1.0 mm (**12–17, 19**); 0.1 mm (**18**).

#### Elevation range.

From slightly above 200 m to slightly above 500 m.

#### Period of activity.

Whole of August.

#### Habitat.

**Figure 20.**
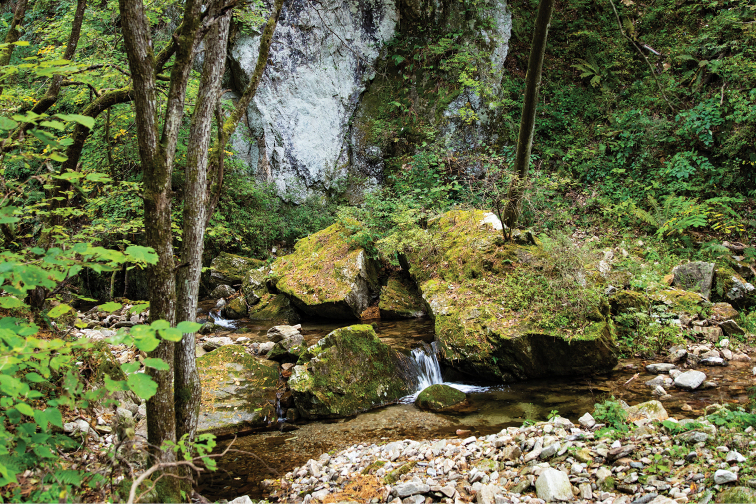
Habitat of Hexatoma (Eriocera) ilwola Podenas, sp. nov., KNU experimental forest.

Sandy and rocky margins of medium–sized mountainous rivers covered with deciduous forest and scarce grassy vegetation (Fig. 20).

#### Distribution.

Korean Peninsula.

#### Remarks.

*Hexatomailwola* sp. nov. is most similar to *H.aequinigra* Alexander, 1934b, which is described and known only from the southern part of the Far East of Russia. *Hexatomaaequinigra* was described from the female, which is distinctly bigger than that of *H.ilwola* sp. nov. *Hexatomaaequinigra* has dense and long pubescence on head and thorax, while it is short and scarce in *H.ilwola* sp. nov. *Hexatomaaequinigra* has dark brown basal antennomeres, which are paler in *H.ilwola* sp. nov. *Hexatomaaequinigra* has pale yellow halter with dark brown knob, while the halter of *H.ilwola* sp. nov. is grayish brown with dark brown knob. Abdominal sternites of *H.aequinigra* are dark brown, but widely yellowish in *H.ilwola* sp. nov. Another similar species is *H.sachalinensis*, which is also known from the Far East of Russia, but it has a brownish black rostrum, dark brown femora, and brownish black tibiae. The rostrum of *H.ilwola* sp. nov. is brown, the legs yellow to brownish yellow. Unfortunately, the male of *H.aequinigra* is unknown and the male terminalia of *H.sachalinensis* have not been illustrated, thus comparison of the structure of male terminalia is not possible at the moment.

### ﻿ Hexatoma (Eriocera) masakii

Taxon classificationAnimaliaDipteraLimoniidae

Alexander, 1934

443B53DE-3351-5170-8C42-5BF4CF262F75

Figs 21–25 , 61

Hexatoma (Eriocera) masakii Alexander 1934b: 48, pl. 1, fig. 18.

#### Type material examined.

***Holotype***, male (pinned, antennae, legs and wing slide mounted), **South Korea**, Suigen, Chosen, 14 August 1930, I. Tabashi leg. (USNM).

#### Other examined material.

**South Korea**, 7 males (pinned), #26, Central National Forest, 18 mi. NE of Seoul, 37°44.89'N, 127°17.62'E, alt. 110 m, 14 August 1954, G. W. Byers leg. (SMEK, USNM).

#### Description.

**Figures 21–25.**
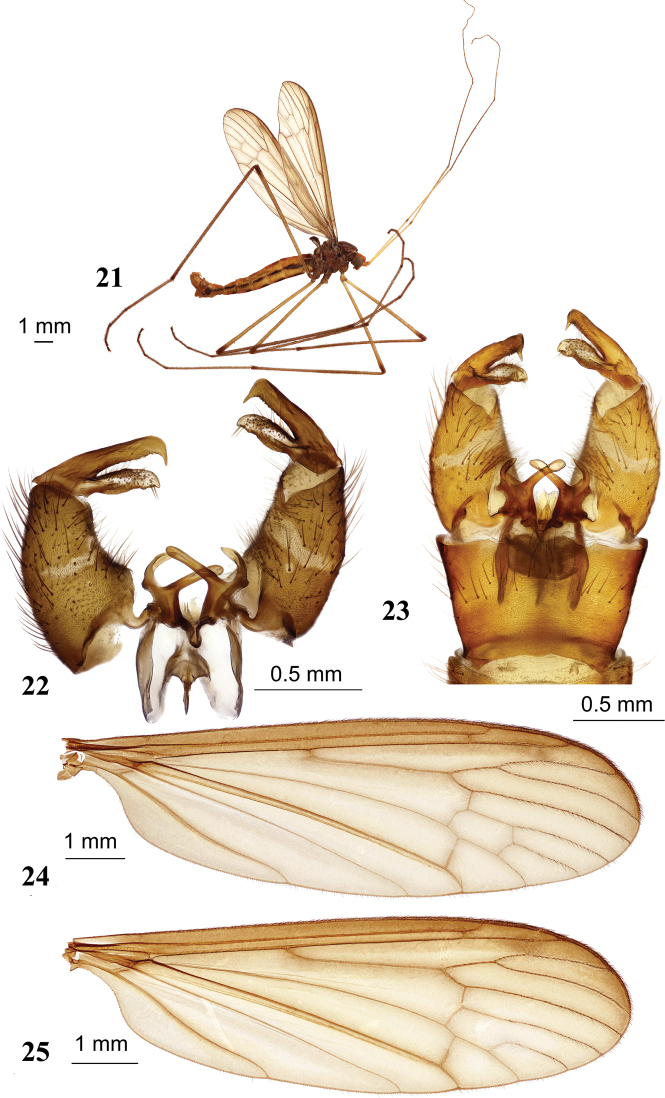
Hexatoma (Eriocera) masakii Alexander, 1934 **21** male, lateral view **22** male genitalia with ninth segment removed, dorsal view **23** male genitalia, dorsal view **24** male wing **25** male wing variation with open discal cell. Scale bars: 1.0 mm (**21, 24, 25**); 0.5 mm (**22, 23**).

***Body***.Thorax brown, head, and abdomen orange-yellow (Fig. 21). Male body length 9.5–11.5 mm, wing length 9.0–10.2 mm.

***Head*.** Orange-yellow, narrowly grayish along posterior eye margin, sparsely covered with short erect brown setae. Vertex with distinct uniformly pale orange-yellow tubercle. Eyes widely separated in male, distance between them at base of antennae nearly the same as length of both basal antennomeres. Male antenna 8-segmented, 16.0–19.5 mm long, ~ 2× as long as wing (Fig. 21). Scape short and wide, approximately as long as wide, 3× as long as pedicel, nearly cylindrical, brownish yellow, covered with few very short brown setae. Pedicel small, subglobular, brownish yellow. Basal flagellomere very long, cylindrical, pale yellow with narrowly dark brown apex. Second flagellomere brownish yellow, darker at base and apex. Remaining flagellomeres brown. Apical segment elongate, subcylindrical, more than twice as short as preceding. All flagellomeres covered with dense erect whitish pubescence and scattered short dark brown setae, length of which less than pubescence on two basal flagellomeres, slightly exceeds length of pubescence on base of third flagellomere, and 2–3× longer on remainder of flagellum. Rostrum orange-yellow. Palpus and mouth parts dark brown.

***Thorax*.** Cervical sclerites and pronotum dark brown. Prescutum blackened. Presutural scutum and prescutum semi-polished dark brown, covered with scattered short brown setae and very sparse brownish pruinosity, longitudinal stripes missing. Tubercular pits missing, pseudosutural fovea distinct. Dorsopleural membrane yellow frontally. Postsutural scutum with each lobe blackish, area between lobes polished dark brown. Scutellum brown, sparsely dusted with brownish pruinosity. Mediotergite brown, darkened posteriorly. Pleuron brown, very sparsely dusted with gray. Episternum bare, setoseless, ventral margin of katepisternum blackish. Meron well developed, second and third pairs of legs staying apart. Wing (Figs 24, 25) iridescent, brownish, with brown frontal margin. Brown area extends through costal area, stigma and reaches vein R_4_. Indistinct darkenings surrounding cord and distal margin of discal cell. Veins brown. Venation: Sc very long, reaching wing margin slightly before branching point of R_2+3_ and R_4_, sc-r shortly beyond branching point of Rs. Radial sector long, nearly straight, slightly arched at base. Free end of R_1_ longitudinal, R_2_ twice its own length beyond branching point of R_2+3_ and R_4_. R_3_ and R_4_ slightly diverging at wing margin, cell r_3_ with long stem, which is twice as long as R_2+3_. Cross-vein r-m distinct, transverse, at base of discal cell. Discal cell nearly twice as long as wide, sometimes open due to reduction of cross-vein m-m (Fig. 25). Cross-vein m-cu at ~ 1/3 of discal cell. Anal vein long, slightly sinuous, apex reaching wing margin slightly before the level of Rs base. Anal angle wide, posterior margin widely rounded. Halter black with pale base. Length of male halter 1.5–1.7 mm. Coxae brown to dark brown, fore coxa yellowish postero–ventrally. Fore trochanter yellowish, middle and hind trochanters yellowish dorsally, brownish ventrally. Femur brownish yellow with pale yellow base and conspicuous black apical ring. Tibia brownish yellow with narrowly infuscate apex. Basal tarsomere brownish with yellow base, remainder of tarsus brown to dark brown or black, covered with long dense dark brown setae. Tibia of fore leg with single apical spur, tibiae of middle and hind pairs of legs with two apical spurs each. Male femur I: 5.2–6.5 mm long, II: 5.0–6.0 mm, III: 5.9–6.0 mm, tibia I: 6.0–8.3 mm, II: 6.4–6.5 mm, III: 7.7–8.0 mm, tarsus I: 7.2–8.5 mm, II: 5.2–6.3 mm, III: 4.5–5.8 mm. Claw simple, without spines.

***Abdomen*.** Abdominal segments orange yellow. Tergite laterally narrowly blackened, with paired transverse suture at ~ 1/3 of length. Sternite with lateral margin narrowly blackened and with longitudinal spot in the middle. Eight sternite without black spot in the middle. Lateral and ventral abdominal lines interrupted at posterior margins of segments. Whole ninth segment compact, making genital ring, yellow dorsally, pale brown ventrally. Male genitalia (Figs 22, 23) brownish yellow to pale brown. Epandrium wider than long, posterior margin with two low wide lobes separated by shallow emargination. Gonocoxite twice as long as wide, slightly wider at base, without additional lobes. Outer gonostylus long and narrow, sclerotized, with sharp apical spine turned mesally, inner margin finely serrated. Inner gonostylus long, fleshy, setose. Paramere with two long narrow arms. Aedeagus simple, short, and straight, apex bifid. Anterior apodeme long and narrow, but extending forward less than lateral margins of aedeagal sheath.

#### Elevation range in Korea.

Slightly above 100 m.

#### Period of activity in Korea.

Middle of August.

#### Habitat.

Unknown.

#### General distribution

(Fig. 61). Endemic to South Korea (erroneously listed for North Korea by Oosterbroek (2022)). May be extinct due to urban development; not one specimen was found in the tens of thousands we collected. However, it is difficult to collect *Hexatoma* adults: you need to be at the right place and at the right time to catch them or to see them swarming.

### ﻿ Hexatoma (Eriocera) pernigrina

Taxon classificationAnimaliaDipteraLimoniidae

Alexander, 1938

59256003-A673-5F27-8FD8-DFF135E88EFA

Figs 26–32 , 62

Hexatoma (Eriocera) pernigrina Alexander 1938: 159, pl. 1, fig. 21.

#### Type material examined.

***Holotype***, male (pinned), **North Korea**, Ompo, alt. 140 m, 14 June 1937, A. Y. Yankovsky leg. (USNM). ***Paratypes***: **North Korea**, 1 male, 1 female (on same pin as holotype), 1 male, 5 females (pinned, antenna, hind leg, wing and terminalia of male slide mounted), topotypic (USNM).

#### Other examined material.

**North Korea**, 6 males, 2 females (pinned), Ompo, alt. 90 m, 9 June 1937, A. Y. Yankovsky leg. (USNM); 1 male, 1 female (pinned), Ompo, alt. 50 m, 28 May 1938, A. Y. Yankovsky leg. (USNM); 1 male (pinned), Chonsani, alt. 920 m, 22 June 1940, A. Y. Yankovsky leg. (USNM); **South Korea**, 1 male, 1 female (pinned), #12, Hwy. #20, 8 mi. SW of Kangnung, 37°42.00'N, 128°47.00'E, alt. 590 m, 8 June 1954, G. W. Byers leg. (SMEK, USNM); 1 female (pinned), Gyeonggi–do, Paju–si, Aengmubong, 37°45.46'N, 126°55.65'E, alt. 550 m, 6 June 1973, Yuseok Kim leg. (KU); 1 male (pinned), Gyeonggi–do, Pocheon–si, Soheul–eup, Gwangneung Forest, 37°45.05'N, 127°09.70'E, alt. 180 m, 10 June 1973, Okjin Lee leg. (KU); 1 male (pinned), Seoul, Mt. Suraksan, 37°41.79'N, 127°04.93'E, alt. 570 m, 4 June 1974 (KU); 1 male (in ETOH), Gyeongsangbuk–do, Yeongju–si, Punggi–eup, Samga–ri (downstream), Mt. Sobaeksan, 36°55.28'N, 128°30.33'E, alt. 400 m, 14 May 2000 (KU); 1 female (in ETOH), Gyeongsangbuk–do, Bonghwa–gun, Mt. Seondalsan, 37°02.38'N, 128°42.55'E, alt. 1220 m, 5 September 2000 (KU); 2 females (in ETOH), same collection data as for preceding, 4 July 2001 (KU); 1 male, 1 female (in ETOH), same collection data as for preceding, 15 September 2001 (KU); 1 male, 1 female (in ETOH), Jeollabuk–do, Namwon, Sannae–myeon, Buun–ri, Namwonsi Sannaemyeon Baemsagol, 35°21.21'N, 127°34.95'E, alt. 830 m, 27 May 2009, S. W. Jung leg. (KU); 1 male (in ETOH), Gangwon–do, Jeongseon–gun, Imgye–myeon, Dojeon–ri, 37°32.15'N, 128°54.17'E, alt. 760 m, 24 May – 23 June 2011 (1), H.–W. Byun et al. leg., Malaise trap (NIBR); 1 female (in ETOH), Gangwon–do, Pyeonchang–gun, Odaesan NP, 37°44.26'N, 128°35.50'E, alt. 730 m, 22 June 2012 (03), S. Podenas leg. (NIBR); 3 males, 2 females (pinned), 7 males, 6 females (in ETOH), Jeollanam–do, Gurve, Masan–myeon, Hwangjeon–ri, 35°14.62'N, 127°29.38'E, alt. 100 m, 8 May 2013 (1), S. Podenas, H.–W. Byun leg. (NIBR); 20 males, 15 females (in ETOH), Gyeonggi–do, Gapyeong–gun, Buk–myeon, Jeokmok–ri, 37°58.61'N, 127°26.59'E, alt. 310 m, 22 May 2014, D.–G. Kim, M.–D. Baek, H.–D. Gang, Ch. Uy leg. sweeping (KU); 1 male, 2 females (in ETOH), Gyeonggi–do, Gapyeong–gun, Buk–myeon, Jeokmok–ri, Garim–gyo (Br.), GERC–H, 37°58.55'N, 127°26.49'E, alt. 310 m, 24–30 May 2015, Y. J. Bae leg., Malaise trap (KU); 1 male (in ETOH), Gyeonggi–do, Gapyeong–gun, Buk–myeon, Jeokmok–ri, Garim–gyo (Br.), GERC–F, 37°58.55'N, 127°26.49'E, alt. 310 m, 7–13 June 2015, Malaise trap (KU); 2 males, 1 female (in ETOH), Gyeonggi–do, Gapyeong–gun, Buk–myeon, Jeokmok–ri, Garim–gyo (Br.), GERC–G, 37°58.55'N, 127°26.49'E, alt. 310 m, 6–12 May 2015, Y. J. Bae leg., Malaise trap (KU); 4 females (pinned), Gyeongsangbuk–do, Gyeongju–si, Yangbuk–myeon, Janghang–ri, 35°45.74'N, 129°21.84'E, alt. 330 m, 28 May 2016 (1), S. Podenas, H. M. Baek leg. (NIBR); 1 female (pinned), Jeollanam–do, Gurye–gun, Toji–myeon, Naeseo–ri, Piagol valley, 35°16.31'N, 127°34.29'E, alt. 490 m, 3 June 2016 (02), S. Podenas leg. (NIBR); 1 female (pinned), Jeollanam–do, Gurye–gun, Toji–myeon, Naeseo–ri, Piagol valley, 35°16.40'N, 127°34.15'E, alt. 550 m, 3 June 2016 (3), S. Podenas leg. (NIBR); 2 males (pinned), 1 female (in ETOH), Gyeonggi–do, Yangpyeong, Cheongun–myeon, Dowon–ri, 37°32.70'N, 127°47.69'E, alt. 220 m, 29 May 2017, S. Podenas leg., at light (NIBR).

#### Description.

**Figures 26–32.**
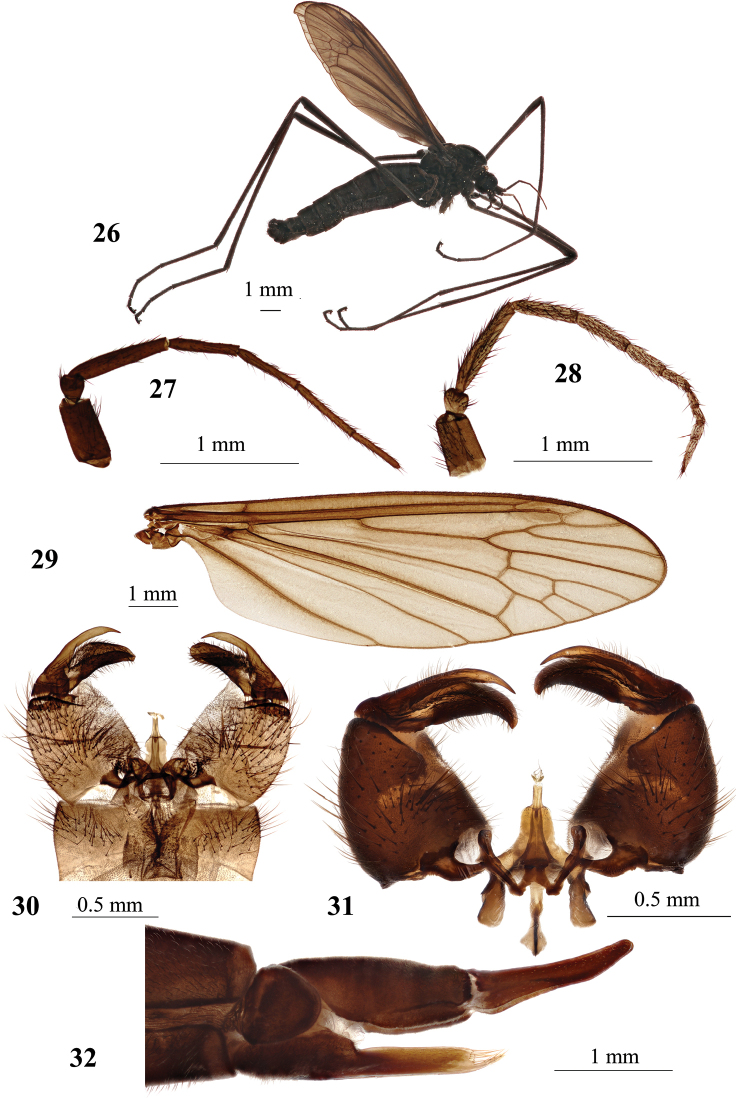
Hexatoma (Eriocera) pernigrina Alexander, 1938 **26** male, lateral view **27** male antenna **28** female antenna **29** male wing **30** male genitalia, dorsal view **31** male genitalia with ninth segment removed, dorsal view **32** ovipositor, lateral view. Scale bars: 1.0 mm (**26–29, 32**); 0.5 mm (**30, 31**).

***Body*** coloration opaque black (Fig. 26). Body length of male 11.5–12.0 mm, female 16.3–19.8 mm, wing length of male 10.0–11.0 mm, female 10.6–15.0 mm.

***Head*.** Opaque black dorsally, dull black ventrally, sparsely covered with erect black setae. Vertex with small tubercle. Eyes widely separated in both sexes, distance between them at base of antennae nearly the same as length of scape. Antenna black at base, turning dark brown towards apex, 7-segmented in male (Fig. 27) (some specimens with fissure in the middle of the last segment, thus antenna looks 8-segmented), 2.6–4.0 mm long, extending to approximately middle of prescutum if bent backward. Female antenna (Fig. 28) 3.0–4.0 mm long. Scape elongate, nearly cylindrical, 1.7× longer than wide, 3× as long as pedicel, pedicel widened distally, bearing a few setae. Flagellomeres elongate, sub–cylindrical, narrower towards apex of antenna, covered with sparse short setae, length of which approximately as width of respective segments. Length of I–V flagellomeres decreases in all specimens, but length ratio varies individually (1.00/0.65 ± 0.02/0.56 ± 0.07/0.41 ± 0.09/0.26 ± 0.04) (mean ± standard deviation). Rostrum black, semi–polished, with few long apical setae. Palpus and mouth parts black.

***Thorax*.** Cervical sclerites and pronotum black. Prescutum and presutural scutum opaque black with four semi–polished stripes, areas between stripes covered with dense short setae. Tubercular pits missing, pseudosutural fovea black, semi-polished. Postsutural scutum with each lobe black covered with grayish pruinosity, area between lobes polished-black. Scutellum dull black with narrow transverse wrinkles. Mediotergite black, laterally covered with grayish pruinosity. Pleuron black, sparsely dusted with gray. Wing (Fig. 29) dark brown, slightly iridescent, with blackish costal area. Stigma same color as darkening along frontal wing margin. Veins dark brown. Venation: humeral vein just slightly before arculus, Sc very long, reaching wing margin slightly beyond branching point of R_2+3_ and R_4_, sc-r shortly before branching point of R_2+3_ and R_4_. Rs long, slightly arched at base. Free end of R_1_ short and oblique, R_2_ 3× its own length before apex of R_1_. R_3_ and R_4_ diverging, cell r_3_ with long stem, which slightly exceeds m-cu in length. Cross-vein r-m distinct, transverse, at base of discal cell. Length of discal cell slightly more than twice its width. Cross-vein m-cu slightly before middle of discal cell. Anal vein long, slightly concave at middle, apex slightly beyond the level of Rs base. Anal angle wide, posterior margin widely rounded. Halter black throughout. Length of male halter 1.4–1.7 mm, that of female 1.5–1.7 mm. Whole leg, including coxa and trochanter, black. Tibia of fore leg with single apical spur, tibiae of middle and hind pairs of legs with two apical spurs each. Male femur I: 5.0–5.2 mm long, II: 6.4–6.5 mm, III: 6.8–7.5 mm, tibia I: 5.7–6.0 mm, II: 5.8–6.0 mm, III: 6.8–7.0 mm, tarsus I: 6.0–6.2 mm, II: 5.5–5.8 mm, III: 5.5–6.0 mm. Female femur I: 4.3–6.5 mm long, II: 4.7–5.0 mm, III: 8.0–9.0 mm, tibia I: 4.7–5.2 mm, II: 2.7–4.4 mm, III: 5.2–6.0 mm, tarsus I: 5.0–5.7 mm, II: 3.5–4.4 mm, III: 3.5–6.0 mm. Claw with subbasal spine.

***Abdomen*.** Abdominal segments black with narrowly grayish posterior margin. Posterior segments dusted with gray, more densely on sternites. Tergites with paired transverse sutures frontally. Male terminalia (Figs 30, 31) black, narrower than pregenital segments. Epandrium wider than long, posterior margin with two short blunt–apexed lobes separated by wide but shallow V-shaped emargination. Gonocoxite slightly longer than wide, slightly arched, without additional lobes. Outer gonostylus long and narrow, sclerotized, pointed and slightly arched. Inner gonostylus slightly longer than outer gonostylus, wide, fleshy, setose. Paramere long and narrow, with distal part curved mesally. Aedeagus simple, short, and straight, tip with brush of few setae. Anterior apodeme long with triangullar lobes on both sides, extending far beyond frontal margin of lateral lobes of aedeagal sheath. Ovipositor (Fig. 32) black with distal part of cercus brown. Tenth tergite elongate. Cercus nearly straight, comparatively stout, narrowing distally, apex rounded, ventral margin slightly sinuous. Hypovalva long, parallel-sided, reaching only base of cercus, blunt apex, apical setae longer ventrally than dorsally.

#### Elevation range in Korea.

From less than 50 m to more than 1200 m.

#### Period of activity in Korea.

From beginning of May through to middle of September.

#### Habitat.

Margins of mountainous small- and medium-sized streams densely covered with deciduous forests. Species is attracted to light.

#### General distribution

(Fig. 62). Endemic to the Korean Peninsula.

### ﻿ Hexatoma (Eriocera) pianigra

Taxon classificationAnimaliaDipteraLimoniidae

Podenas sp. nov.

53762E14-7482-51FF-8C06-BB08759B475F

http://zoobank.org/5F5B56FA-5ACA-47B6-97BF-DDCACFDE7513

Figs 33–38 , 63

#### Type material examined

**Figures 33–38.**
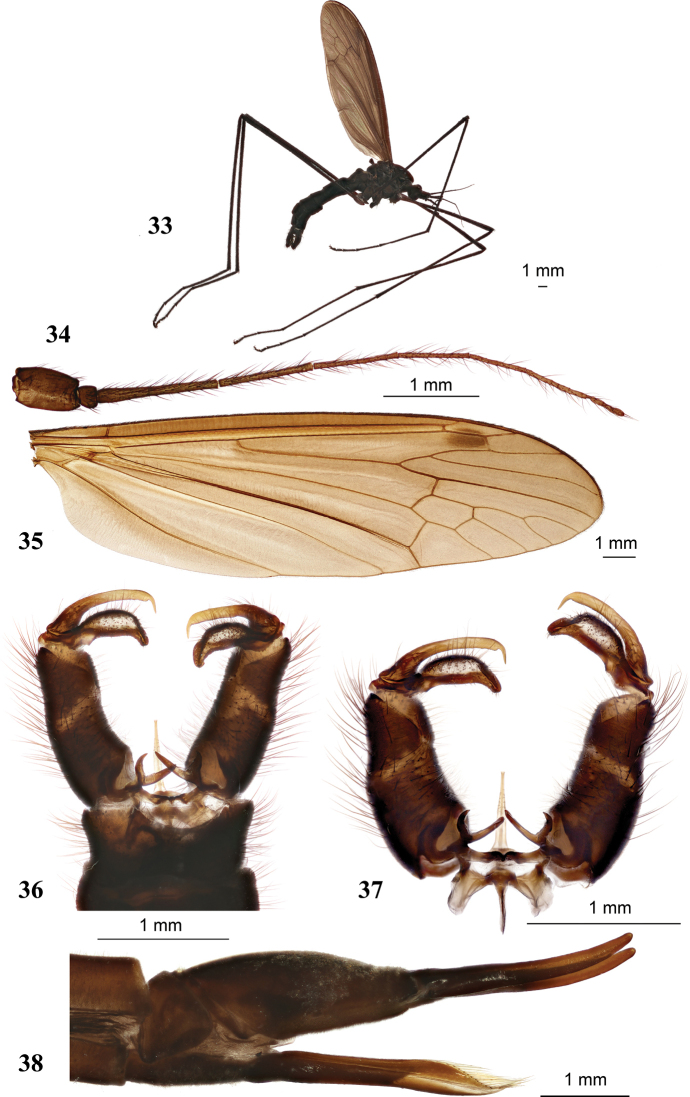
Hexatoma (Eriocera) pianigra Podenas, sp. nov. **33** holotype, male, lateral view **34** female antenna, paratype **35** female wing, paratype **36** male genitalia, dorsal view, holotype **37** male genitalia with ninth segment removed, dorsal view **38** ovipositor, paratype. Scale bars: 1.0 mm.

(Fig. 63). ***Holotype***, male (Fig. 33) (pinned), **South Korea**, Jeollanam–do, Gurye–gun, Toji–myeon, Naeseo–ri, Piagol valley, 35°15.95'N, 127°34.85'E, alt. 450 m, 3 June 2016 (1), S. Podenas leg. (NIBR). ***Paratypes***: **South Korea**, 1 female (pinned), #25, Central National Forest 18 miles NE of Seoul, 14 August 1954, G. W. Byers leg, (USNM); 1 female (pinned), Jeollanam–do, Gurye–gun, Toji–myeon, Naeseo–ri, Piagol valley, 35°15.50'N, 127°34.93'E, alt. 310 m, 29 June 2015 (2), S. Podenas leg. (NIBR); 1 female (pinned), Jeollanam–do, Gurye–gun, Toji–myeon, Naeseo–ri, Piagol valley, 35°16.03'N, 127°34.66'E, alt. 460 m, 27 June 2019 (3), S. Podenas leg. (NIBR).

#### Diagnosis.

Large black species (Fig. 33) with body length 14.0–31.5 mm. Male antenna reaching to base of halter if bent backwards. Prescutum and presutural scutum with three distinct stripes. Wing brown with distinct stigma and darkened costal area. Cell m_1_ present. Legs dark brown to black. Abdomen entirely black. Epandrium of male genitalia with slightly concave posterior margin. Gonocoxite 2.6× longer than wide. Inner gonostylus wide, slightly arched, swollen in the middle. Paramere arched. Aedeagus simple, long, narrow, straight. Ovipositor brown.

#### Etymology.

The species is named after the type locality, the Pia River, and for the black color of the body (= *nigra*).

#### Description.

Body coloration black, semi-polished. Body length of male 14.0 mm, female 26.5–31.5 mm, wing length of male 16.3 mm, female 17.8–21.0 mm.

***Head*.** Black, densely covered with brownish gray pruinosity and scattered short erect black setae. Eyes marginated by narrow whitish gray. Vertical tubercle large, rounded, with indistinct medial groove, concolorous with the rest of the head. Eyes widely separated in both sexes, distance between them at the base of antennae equal to length of scape. Male antenna 7-segmented, 5.2 mm long, reaching to approximately base of halter if bent backwards. Scape large, 2× as long as wide, 4× as long as pedicel, dark brown to blackish, sparsely dusted with brownish. Pedicel wider than long, black. Flagellum entirely black, densely covered with semi–erect black setae. Basal flagellomere longer than both basal antennomeres taken together and slightly longer than second flagellomere, third flagellomere longest, apical segment very small, button-shaped. Female antenna (Fig. 34) 11-segmented, 6.5–7.0 mm long, reaching wing base if bent backwards, entirely black. Rostrum dark brown, dusted with grayish. Palpus and labellum black, dusted with grayish brown pruinosity.

***Thorax*.** Cervical sclerites dark brown dorsally, brown laterally, densely dusted with gray. Pronotum dark brown, postero–lateral angle polished rusty brown. Prescutum and presutural scutum densely dusted with orange–brownish gray, with three distinct stripes, medial stripe laterally semi-polished dark brown, divided along middle with densely dusted area, lateral stripe polished black. Tubercular pits missing, pseudosutural fovea black, semi-polished. Postsutural scutum with each lobe black, sparsely dusted. Area between lobes densely covered with pruinosity. Scutellum dark brown, densely dusted, covered with sparse short erect setae. Mediotergite dark brown densely dusted with grayish brown. Pleuron dark brown, dusted with grayish brown. Wing (Fig. 35) brown, dark brown along frontal margin and along cubital vein, iridescent, stigma dark brown, elongate, but not very distinct because of dark background. Veins brown to dark brown. Macrotrichiae on distal veins very scarce, nearly missing. Venation: humeral vein slightly before arculus, Sc very long, reaching wing margin distinctly beyond branching point of R_2+3_ and R_4_, sc-r at branching point of R_2+3_ and R_4_. Rs long, slightly arched at base. Free end of R_1_ elongate, R_2_ 2× its own length before apex of R_1_. R_3_ and R_4_ diverging, cell r_3_ with long stem, which is approximately as long as m-cu. Cross-vein r-m distinct, transverse, at base of discal cell. Discal cell slightly more than 2× as long as wide. Cell m_1_ approximately as long as its stem or slightly shorter. Cross-vein m-cu at ~ 1/4 of discal cell length. Anal vein long, slightly sinuous, apex beyond the level of Rs base. Anal angle wide, posterior margin widely rounded. Entire halter dark brown except pale brown base of stem. Length of male halter 2.0 mm, that of female 2.0–2.2 mm. Coxa dark brown densely dusted, trochanter dark brown, femur dark brown to black with narrowly brownish base, remainder of leg dark brown to black. Tibia of fore leg with single apical spur, tibiae of middle and hind pairs of legs with two apical spurs each. Legs covered with short dense semi-erect setae. Male femur I: 9.0 mm long, II: 11.5 mm, III: 13.0 mm, tibia I: 11.7 mm, II: 10.5 mm, III: 13.7 mm, tarsus I: 11.8 mm, II: 9.2 mm, III: 8.7 mm. Female femur I: 10.0–10.5 mm long, II: 12.0–12.5 mm, III: 12.0–14.0 mm, tibia I: 11.2–11.5 mm, II: 11.0 mm, III: 10.5–15.5 mm, tarsus I: 11.5–11.7 mm, II: 8.7–9.0 mm, III: 8.4–8.5 mm. Claw rusty brown with subbasal spine.

***Abdomen*.** Male abdomen black, semi-polished, dusted with brownish pruinosity, covered with erect sparse whitish setae, longer on sternites, shorter on tergites. Posterior margins of tergites and sternites narrowly grayish. Tergites with two pairs of transverse sutures. Female abdomen dark brown, coloration of sternites slightly varies individually from brown to dark brown, in some females basal sternites pale brown, in some seventh sternite pale brown to yellowish brown. Male terminalia (Figs 36, 37) dark brown, gonocoxites rusty medially, outer gonostylus pale brown. Epandrium wider than long, posterior margin slightly concave. Gonocoxite elongate, 2.6× longer than wide. Two pairs of long narrow gonostyli. Outer gonostylus sclerotized, point–apexed and slightly arched, apical part at right angle to longitudinal axis of gonostylus. Inner gonostylus approximately as long as outer gonostylus, wide, fleshy, and setose, slightly arched, swollen at middle. Paramere with two long and narrow arms, dorsal arm slightly arched, ventral nearly straight, longer than dorsal. Aedeagus very long, narrow, simple, straight. Anterior apodeme long and narrow, extending forward beyond lateral lobes of aedeagal sheath. Ovipositor (Fig. 38) brown. Tenth tergite elongate, blackish basally, brownish distally and laterally. Cercus round–apexed, distal part slightly raised upwards, brown, polished, blackened at base. Hypovalva long, parallel-sided to approximately middle, slightly swollen subapically, reaching to ~ 1/3 of cercus, pointed apex, with long setae along dorsal margin distally.

#### Elevation range.

300–500 m.

#### Period of activity.

From beginning of June through to mid–August.

#### Habitats.

**Figure 39.**
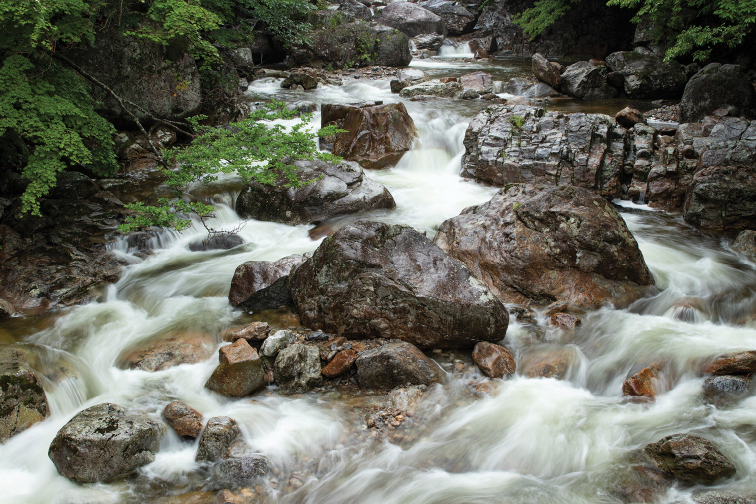
Type locality of Hexatoma (Eriocera) pianigra Podenas, sp. nov., Jirisan National Park, Piagol valley.

Mountainous medium–sized rivers with sandy or fine gravel covered margins surrounded by dense mixed forests (Fig. 39).

#### Distribution.

South Korea.

#### Remarks.

There are a few black *Eriocera* species with cell m_1_ recorded from territories close to the Korean Peninsula, but some of them have unknown males. Among those with males described, the male terminalia are usually unstudied and separation of them is mostly based on external features such as coloration or comparative length of separate structures. *Hexatomaaequinigra* Alexander, 1934b is known only from the female, the size and general appearance of which is similar to that of *H.pianigra* sp. nov., but the species can be easily separated by leg coloration, the femur of *H.pianigra* sp. nov. is black with a narrowly brownish base, while that of *H.aequinigra* is yellow with only the tip blackened. *Hexatomaatripes* Alexander, 1934b is also described from the female only, the measurements of which are also close to *H.pianigra* sp. nov., but the halter has a yellow stem and blackened knob, while the halter of *H.pianigra* sp. nov. is entirely black. The male of *H.issikii* (Alexander, 1928) is somewhat larger than *H.pianigra* sp. nov., it has a yellow mesonotum, a pleuron with a broad stripe and a yellow halter with only the knob blackened. All these structures are completely black in *H.pianigra* sp. nov. *Hexatomalygropis* (Alexander, 1920) is a somewhat larger species with a velvety black body, *H.pianigra* sp. nov. is semi-polished with a sparse cover of pruinosity. *Hexatomanigrotrochanterata* (Alexander, 1932) is similar in size to *H.pianigra* sp. nov., but both species can be easily separated based on leg coloration. The femur of *H.pianigra* sp. nov. is black, while that of *H.nigrotrochanterata* is yellow with only the apical part blackened. *Hexatomapieliana* Alexander, 1940 is described from the female with the male unknown, but it can be easily separated from *H.pianigra* sp. nov., because it has yellow legs and orange yellow abdominal sternites. Males of *H.imperator* Alexander, 1953b, *H.jozana* (Alexander 1924), *H.longeantennata* (Lackschewitz, 1964), *H.pallidibasis* Alexander, 1953a, *H.sachalinensis* (Alexander, 1924), *H.stricklandi* (Edwards, 1921) and *H.superba* Savchenko, 1976 have long antennae, which are at least close to the body length, but usually a few times that. Two other similar species, *H.fumidipennis* (Alexander, 1927) and *H.morula* Alexander, 1923, are described from Sichuan, China. *Hexatomafumidipennis* is dull gray and bigger than *H.pianigra* sp. nov. with a clear wing except a distinctly darkened costal area. *Hexatomamorula* generally looks more like *H.pianigra* sp. nov., but is much smaller with a wider wing and distinct differences in wing venation, especially the long vein Sc reaching slightly beyond R_2_, while it is just slightly beyond the branching point of R_2+3_ and R_4_ in *H.pianigra* sp. nov.

### ﻿ Hexatoma (Eriocera) serenensis

Taxon classificationAnimaliaDipteraLimoniidae

Podenas sp. nov.

EAA85C5A-CF97-594D-8A0D-7B00DE3C7BFE

http://zoobank.org/A5691FAE-C487-460B-9A01-59148A694183

Figs 40–46 , 64

#### Type material examined

(Fig. 64). ***Holotype***, male (Fig. 41) (pinned), **North Korea**, Seren Mts., alt. 610 m, 18 July 1938, A. M. Yankovsky leg. (USNM). ***Paratypes*: North Korea**,1 male, 1 female (pinned) (Fig. 45), topotypic (USNM).

#### Diagnosis.

Large crane fly with body length 16.0–23.5 mm. Body dark brown, densely dusted with gray. Male antenna ~ 3× as long as the whole body. Prescutum and presutural scutum with four stripes. Wing clear with dark stigma. Cell m_1_ present. Abdominal tergites dark brown, lateral margins brownish orange. Male gonocoxite nearly 3× as long as width at base. Inner gonostylus nearly parallel-sided, distal part smoothly narrows to blunt apex. Aedeagus simple, short, straight. Paramere with long narrow branches widely separate at base. Ovipositor brownish orange.

#### Etymology.

Species is named after type locality, the Seren mountains.

#### Description.

***Body*** coloration dark brown densely dusted with gray pruinosity. Body length of male 16.0–16.5 mm, female 23.5 mm, wing length of male 19.0–20.5 mm, female 20.0 mm.

***Head*.** Gray due to dense pruinosity, densely covered with long yellowish erect setae in male, pubescence less dense and distinctly shorter in female. Vertical tubercle large, rounded, dark brown, densely covered with long yellowish erect setae. Area between bases of antennae and tubercle brownish yellow. Eyes widely separated in both sexes, distance between them at base of antennae equals length of scape and pedicel taken together. Male antenna 7-segmented, 50.0–50.3 mm long, ~ 3× as long as entire body (Fig. 41). Scape large, 1.7× long than wide, dark brown at base, brownish orange at distal half, sparsely dusted with gray. Pedicel short and wide, more than twice as wide as long, brownish yellow. Flagellum entirely dark brown, flagellomeres with two parallel lines of short spines medially. Basal flagellomere longer than head and thorax taken together, second–fourth segments getting longer, length ratio of first to fourth segments 1.0: 1.5: 2.4: 3.0, apical segment very small button-shaped. Female antenna 11-segmented, 5.7 mm long, reaching wing base, if bent backwards. Scape 0.68 mm long, brown with dark brown base, dusted with gray, nearly cylindrical. Pedicel 0.16 mm long, cup-shaped, pale brown. Flagellomeres elongate, sub-cylindrical, covered with dark brown setae, single row of spine-shaped setae only on basal flagellomere, length of 1–5 flagellomeres respectively 1.07 mm, 0.73 mm, 0.68 mm, 0.64 mm, 0.56 mm. Rostrum brown, paler ventrally. Palpus and mouth parts dark brown.

***Thorax*.** Cervical sclerites dark brown, densely dusted with gray dorsally. Pronotum gray, lateral angle brownish orange-yellow. Prescutum and presutural scutum brownish gray, pale gray laterally with four distinct dark brown stripes (Fig. 42), covered with dense long erect yellowish setae dorsally, whitish laterally in male, pubescence less dense and distinctly shorter in female. Area separating medial stripes as wide as stripe itself. Tubercular pits very small, close to each other at frontal margin of sclerite, pseudosutural fovea comparatively small, orange posteriorly, dark brown frontally. Postsutural scutum with each lobe bluish gray with widely dark brown central area. Area between lobes gray. Scutellum dark brown, dusted with gray, more densely along posterior margin, covered with very long and dense whitish erect setae posteriorly and laterally. Mediotergite dark brown, gray pruinosity denser frontally, fronto–lateral corner yellowish. Pleuron whitish gray, silvery brown dorsally, densely covered with long erect yellowish setae. Wing (Fig. 43) brownish, darker along frontal margin and along cubital vein, iridescent, with distinct dark brown elongate stigma. Veins brown. Macrotrichiae on distal veins very scarce, nearly missing. Venation: humeral vein at same level as arculus, Sc very long, reaching wing margin distinctly beyond branching point of R_2+3_ and R_4_, sc-r at branching point of R_2+3_ and R_4_. Rs long and nearly straight, slightly arched at base. Free end of R_1_ elongate, R_2_ 3× its own length before apex of R_1_. R_3_ and R_4_ diverging, cell r_3_ with long stem, which slightly exceeds m-cu in length. Cross-vein r-m distinct, transverse, at base of discal cell. Discal cell twice as long as wide. Cross-vein m-cu at ~ 1/4 of discal cell length. Anal vein long, slightly concave at middle, apex far beyond the level of Rs base. Anal angle wide, posterior margin widely rounded. Stem of halter orange-yellow, knob dark brown. Length of male halter 2.0–2.1 mm, that of female 2.1 mm. Coxa gray covered with dense long erect setae, trochanter brown dusted with gray. Fore and middle femora of male yellow before middle, brown to dark brown beyond middle, posterior femur dark brown with ~ 1/3 yellow at base. Femur of female dark brown with only ~ 1/4 yellow at base, yellow area subequal on all legs. Tibia brown with narrowly yellowish base and dark brown apex. Tibia of fore leg with single apical spur, tibiae of middle and hind pairs of legs with two apical spurs each. Tarsal segments brown to dark brown. Male femur I: 7.0 mm long, II: 8.5–9.3 mm, III: 14.0–15.0 mm, tibia I: 12.5 mm, II: 10.0–10.1 mm, III: 13.8–14.5 mm, tarsus I: 14.3 mm, II: 6.3 mm, III: 9.0 mm. Female femur I: 8.5 mm long, II: 10.0 mm, III: 13.5 mm, tibia I: 11.0 mm, II: 9.5 mm, III: 14.0 mm, tarsus I: 11.5 mm, II: 9.2 mm. Claw orange with dark brown apex bearing subbasal spine.

**Figures 40–46.**
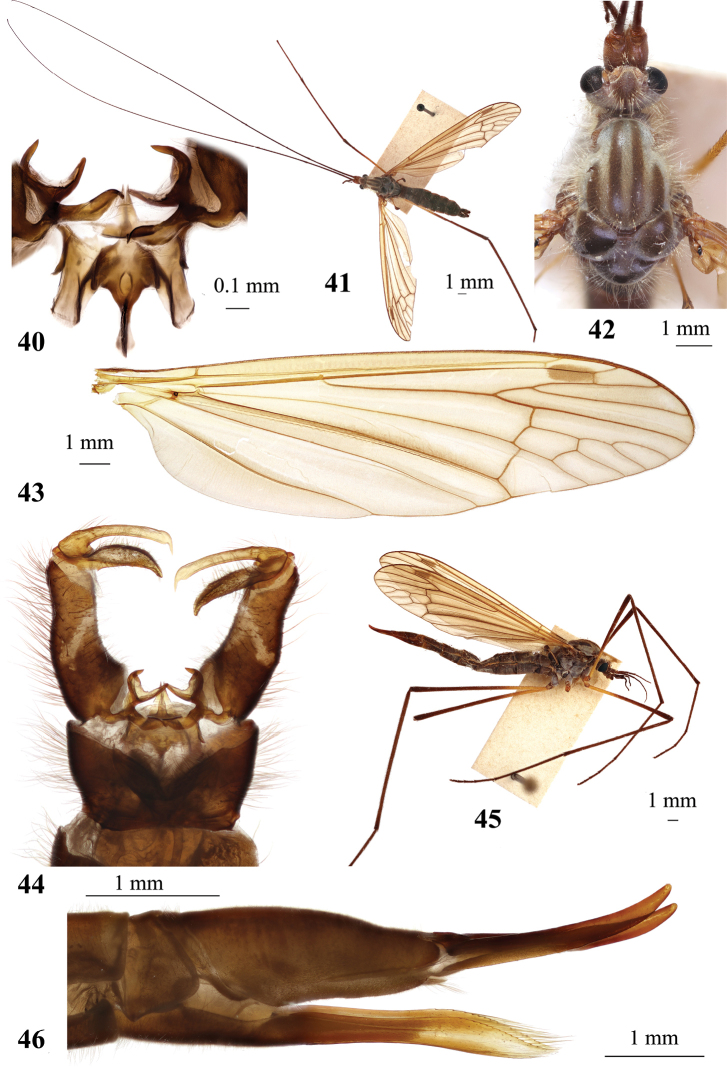
Hexatoma (Eriocera) serenensis Podenas, sp. nov. **40** aedeagal complex, dorsal view, paratype **41** holotype, male, dorsal view **42** head and thorax, dorsal view, holotype, male **43** male wing, paratype **44** male genitalia, dorsal view, paratype **45** female, lateral view, paratype **46** ovipositor, lateral view, paratype. Scale bars: 0.1 mm (**40**); 1.0 mm (**41–46**).

***Abdomen*.** Tergites dark brown with dense cover of gray pruinosity, dusting less intense along middle and posterior margin, covered with long erect yellowish setae. Basal tergite with brownish orange frontal margin, remaining tergites narrowly gray along posterior margin. Lateral margins brownish orange. Second tergite with two distinct pairs of transverse sutures, remaining tergites with second pair less distinct. Sternites dark brown dusted with gray, narrowly orange laterally, each with paired transverse suture at base. Male terminalia (Fig. 44) dark brown, narrower than pregenital segments. Epandrium wider than longer, posterior margin concave. Gonocoxite elongate, nearly 3× as long as width at base, dorsal surface with oblique narrow membranous suture. Two pairs of long narrow gonostyli. Outer gonostylus sclerotized, long, parallel-sided, apex distinctly narrowed and spine-shaped. Inner gonostylus elongate, fleshy and setose, nearly parallel-sided, distal part smoothly narrows to blunt apex. Paramere (Fig. 40) bifid, U-shaped, with long narrow branches widely separate at base. Aedeagus simple, short, and straight, bifid at apex. Anterior apodeme long and narrow, with small lateral lobes on each side, extending forward beyond frontal margin of lateral lobe of aedeagal sheath. Female pregenital segment (Fig. 45) and ovipositor (Fig. 46) orange. Tenth tergite elongate. Cercus slightly darker at base, nearly straight, rounded apex, distal part slightly raised upwards, very apex pale. Hypovalva long, parallel-sided at ~ 2/3 from base, distal part widened and setose, reaching to ~ 1/3 of cercus.

#### Elevation range.

Slightly above 600 m.

#### Period of activity.

Mid-July.

#### Habitat.

Unknown.

#### Distribution.

North Korea, Seren Mountains.

#### Remarks.

*Hexatomaserenensis* sp. nov. is most similar to *H.superba* Savchenko, 1976, which is described and known only from Kunashir Island, Russia (Table 2). *Hexatomaserenensis* sp. nov. is also similar to *H.aequinigra* from Eastern Siberia, but female of *H.aequinigra* has dense and long pubescence on head and thorax, when that is short and scarce in *H.serenensis* sp. nov., femur of *H.aequinigra* female yellow with just distal sixth or less blackened, when femur of *H.serenensis* sp. nov. yellow only at ~ 1/4 from base. Distal wing veins with macrotrichiae nearly missing in *H.serenensis* sp. nov. and *H.superba*, but abundant in *H.aequinigra*. In general, female of *H.serenensis* sp. nov. looks closer to female of *H.superba*, male to *H.aequinigra*. Unfortunately male terminalia of *H.superba* aren‘t described or illustrated, male of *H.aequinigra* unknown, thus comparison of structure of male terminalia isn‘t possible at the moment.

**Table 2.** Comparison of *Hexatomaaequinigra*, *H.superba*, and *H.serenensis* sp. nov.

Character	*H.aequinigra*	*H.superba*	*H.serenensis* sp. nov.
Pubescence of female head	dense and long	scarce and medium-long	medium-long
Vertical tubercle	reddish on either side of midline of vertical tubercle	same color as rest of the head, brownish gray	dark brown, rest of the head gray
Male antenna	–	slightly more than twice as long as body	3.5× as long as body
Pubescence of thorax	dense and long	scarce and medium-long	dense and long
Area separating medial prescutal stripes	slightly wider than the stripes	distinctly narrower than stripes	as wide as stripes
Lateral margin of prescutum	suffused with pale brown	with additional dark spot	uniformly pale gray
Femur	yellow, tip narrowly blackened	basal 1/3 brownish yellow	basal 1/2 brownish yellow
Cell *m_1_*	as long as its stem	as long as 1/2 of its stem	as long as its stem
Abdominal tergites	female dark brown with yellowish brown lateral margin	male uniformly dark brown	male dark brown with brownish orange lateral margin
Female body length (mm)	33	26–27	23.5
Macrotrichiae on distal wing veins	abundant	missing	missing

### ﻿ Hexatoma (Eriocera) stackelbergi

Taxon classificationAnimaliaDipteraLimoniidae

Alexander, 1933

22780C87-2219-5CA0-8B92-C73B5DEAD68D

Figs 47–49 , 65

Hexatoma (Eriocera) stackelbergi Alexander 1933: 152–153, pl. 1, fig. 14; Savchenko 1983: 67 (short note on distribution); 1989: 123 (short note on distribution); Podeniene and Gelhaus 2015: 104–107 (descriptions of larva and pupa), figs 29–38.

#### Type material examined.

***Paratype***, male (antenna, fore leg, and both wings slide-mounted), **Russia**, E. Siberia, Ussuri, Tigrowaja, Suchan distr., 43°15.00'N, 133°00.00'E, [alt. 250 m], 11 June1927, Stackelberg leg. (USNM).

#### Other examined material

(Fig. 65). **North Korea**, 1 female (pinned, fragments of ovipositor in microvial on same pin), Kankyo Nando Puksu Pyaksan, alt. 1830 m, 29 July 1939, A. Yankovsky leg. (USNM).

#### Description.

**Figures 47–49.**
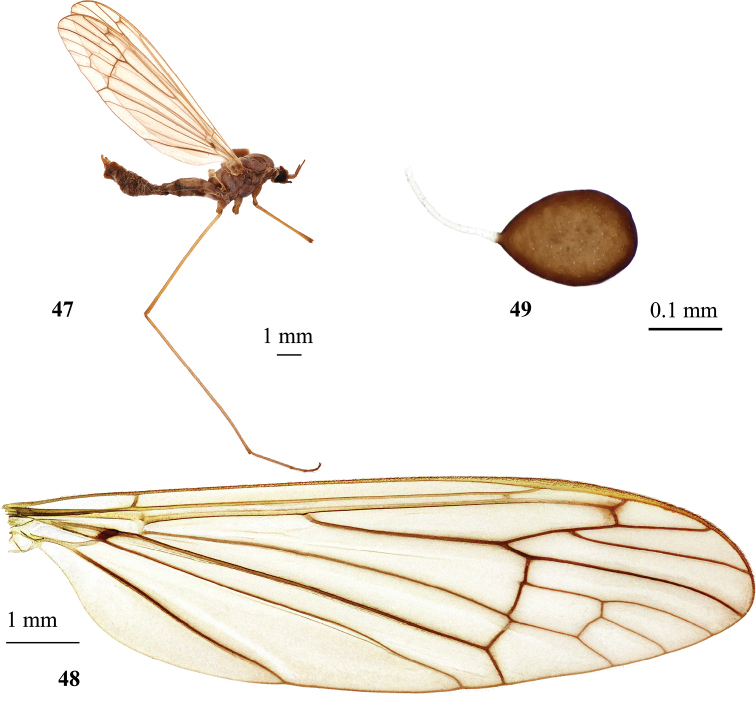
Hexatoma (Eriocera) stackelbergi Alexander, 1933 **47** female, lateral view **48** female wing **49** spermatheca. Scale bars: 1.0 mm (**47, 48**); 0.1 mm (**49**).

***Body*** coloration brownish gray (Fig. 47). Body length of female 8.8 mm, wing length 10.4 mm.

***Head*.** Gray, Brownish gray anteriorly, pale gray posteriorly, covered with short whitish erect setae. Vertical tubercle large, divided by medial grove longitudinally. Eyes widely separated, distance between them at base of antennae approximately equals to length of scape. Basal segments of antenna brown. Scape elongate, 1.6× longer than wide, 2.7× as long as pedicel, darker at base, dusted with gray. Pedicel short, subglobular. Basal flagellomere 1.6× longer than scape, nearly cylindrical, second flagellomere 0.6× as long as basal. Flagellomeres covered with short semi–erect whitish pubescence. Rostrum very short, dark brown, palpus black, labella pale brown.

***Thorax*.** Cervical sclerites pale brown, dusted with gray. Pronotum much wider than long, yellowish brown with gray dusting. Prescutum and presutural scutum gray with three darker brown longitudinal stripes and covered with sparse short whitish setae. Medial stripe separated longitudinally by narrow darker line. Tubercular pits missing, pseudosutural fovea small, pale brown. Postsutural scutum with each lobe gray with indistinct darker brownish spots anteriorly, at middle and posteriorly. Scutellum brownish or yellowish gray with longer yellowish setae along posterior margin. Mediotergite brownish gray, more brownish posteriorly. Pleuron gray with brownish spots where gray pruinosity scarcer. Laterotergite with dense long yellowish setae posteriorly. Wing (Fig. 48) slightly iridescent, subhyaline, with pale grayish tinge, yellowish in costal area and at base. Stigma missing except darkening around R_1_ and R_2_. All veins surrounded by brownish, but no other spots. Veins brown, yellowish at wing base, except distinctly dark brown axillary vein. Macrotrichiae on distal veins missing. Venation: humeral vein slightly before arculus, Sc long, reaching wing margin slightly beyond branching point of Rs, sc-r at or slightly before branching point of Rs. Rs long, slightly arched, short spurred at base in paratype. Free end of R_1_ short and oblique, R_2+3_ just slightly exceeds R_2_ in length. R_3_ and R_4_ diverging, ~ 2.5× as long as its stem. Cross-vein r-m distinct, in alignment with basal deflection of M_1+2_ (base of discal cell). Discal cell slightly more than twice as long as wide. Cross-vein m-cu very slightly beyond base of discal cell (branching point of M). Vein CuP curved at distal part, thus cell cua gets wider towards wing margin. Anal vein long, slightly sinuous, reaching wing margin slightly before or at same level as Rs base. Anal angle wide, posterior margin widely rounded. Halter with stem and knob yellow, base of stem brownish. Length of female halter 1.2 mm. Coxae brown anteriorly, grayish yellow posteriorly, covered with short erect whitish setae. Trochanters obscure yellow. Femur with basal half pale yellow, distal brown. Tibia and tarsus brown to dark brown. Tibia of posterior leg with two apical spurs. Female femur I: 3.0 mm long, III: 5.5 mm, tibia III: 8.0 mm, tarsus III: 3.5 mm. Claw yellowish at base, blackish at apex, with subbasal spine.

***Abdomen*.** Tergites brown, dusted with gray, with one pair of transverse sutures, covered with short yellowish setae. Sternites yellowish brown. Male hypopygium large, black, epandrium wider than long, polished black (Alexander 1933). Ovipositor yellowish brown. Spermatheca (Fig. 49) brown with small paler dots, ovoid.

#### Elevation range.

Above 1800 m in Korea, ca. 250 m in Russia.

#### Period of activity.

Late August in Korea, middle of June in the Far East of Russia.

#### Habitat.

Unknown in Korea. Larvae of this species develop in the bottom gravel of large and medium size rivers. Last instar larvae and pupae can be found in the riparian zone, usually in gravel, sand or under stones in Mongolia (Podeniene and Gelhaus 2015).

#### General distribution.

Far East of Russia and Mongolia. Recorded on the Korean Peninsula for the first time.

#### Remark.

*Hexatomastackelbergi* was known only from three type specimens, all males, listed in Alexander (1933). The male genitalia were not illustrated and we had no possibility to study them. This is the first record not only for Korea, but it is also the first specimen besides the types and the first female. Unfortunately, it has badly damaged terminalia, thus a more detailed study of the ovipositor is not possible at this time.

### ﻿ Hexatoma (Eriocera) ussuriensis

Taxon classificationAnimaliaDipteraLimoniidae

Alexander, 1934

14105E86-8196-5189-A5DD-0E24E6336E67

Figs 50–53 , 66

Hexatoma (Eriocera) ussuriensis Alexander 1934a: 341–343, pl. 1, fig. 22, pl. 4, fig. 49; Savchenko 1983: 68 (short note on distribution); 1989: 123 (short note on distribution); Przhiboro et al. 2009: 221–228 (distribution and habitats); Podeniene and Gelhaus 2015: 107–112 (descriptions of larva and pupa), figs 39–53.

#### Type material examined.

***Paratypes*: Russia**, male (antenna, fore leg, wing, and genitalia slide–mounted), E. Siberia, Ussuri, Bikin, river Bikin, 8 July 1927, Martynov leg. (USNM); 1 specimen, sex unknown (pinned), Ussurian district, river Bikin, station Bikin, 8–9 July 1927, Martynov leg. (USNM); 1 male (pinned), same collection data as for preceding, 9 July 1927 (USNM).

#### Other examined material

(Fig. 66). **North Korea**, teneral male (pinned), Prov. South Pyongan, Pyongyan, Hotel garden, 5 August 1971, No 141, S. Horvatovich et J. Papp leg. (HNHM).

#### Description.

**Figures 50–53.**
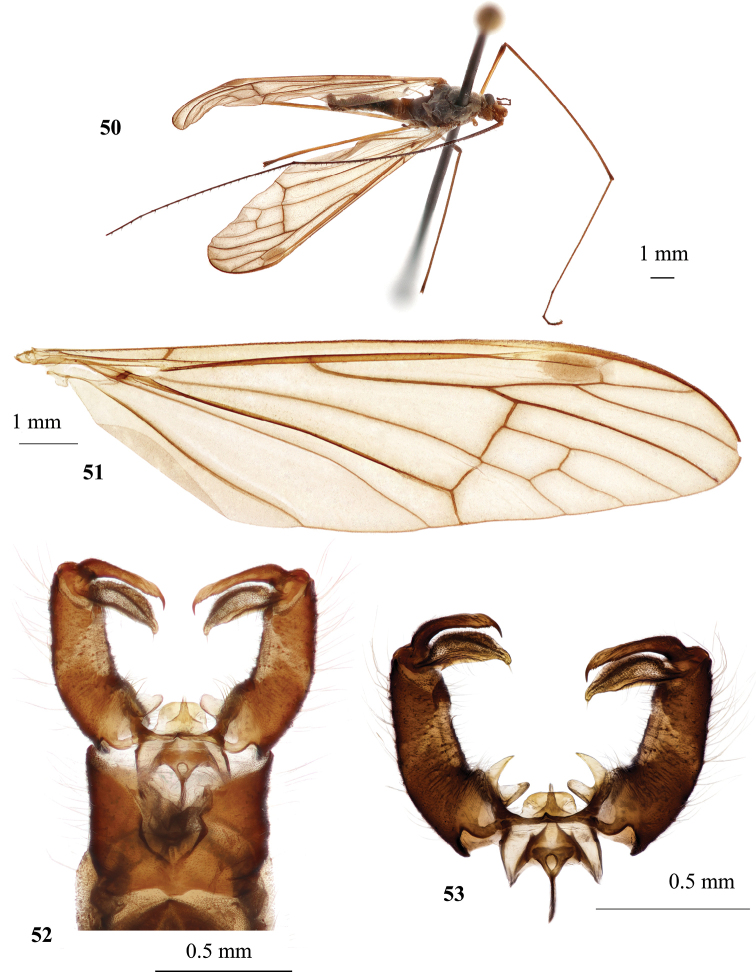
Hexatoma (Eriocera) ussuriensis Alexander, 1934 **50** male, dorsal view **51** male wing **52** male genitalia, dorsal view **53** male genitalia with ninth segment removed, dorsal view. Scale bars: 1.0 mm (**50, 51**); 0.5 mm (**52, 53**).

***Body*** coloration gray to dark brown (Fig. 50). Male body length 7.5 mm, wing length 12.5 mm.

***Head*.** Brownish gray, narrowly pale gray along eye margin, covered with whitish erect setae. Vertical tubercle very large, rounded, brown, dusted with grayish yellow postero-dorsally, without polished black summit mentioned in original description (Alexander 1934a), polished black area also missing in studied topotypic paratypes. Tubercle reaches beyond middle of scape. Eyes widely separated, distance between them at base of antennae equal to length of scape and pedicel taken together. Male antenna 18.9 mm long, distinctly longer than the entire body. Scape very large, elongate, obscure yellow with darkened and dusted dorsal surface. Pedicel very short, ring-shaped, yellow. Flagellum black with base of first flagellomere narrowly brownish. Flagellomeres with two parallel rows of short spines. Basal flagellomere 4.2 mm long, with 19 spines in each row and sparse short whitish pubescence between them, second flagellomere 6.0 mm long with 24 spines, third flagellomere 7.2 mm long with 21 spines. Rostrum brownish yellow with few long erect setae dorsally, palpus dark brown to blackish, labella black.

***Thorax*.** Cervical sclerites brown, dusted with gray. Pronotum much wider than long, dark brown with gray dusting. Prescutum and presutural scutum gray with four distinct dark brown longitudinal stripes and covered with comparatively sparse long whitish setae. Area separating medial stripes slightly narrower than the stripes themselves. Tubercular pits missing, pseudosutural fovea small, polished–brown. Postsutural scutum with each lobe gray with dark brown central area, which also dusted with gray. Area between lobes dark brown anteriorly, pale posteriorly. Scutellum gray with pale fronto-lateral angle. Mediotergite brown, dusted with gray. Pleuron gray, posterior margin of anepimeron with dense long yellowish setae. Wing (Fig. 51) slightly iridescent, with pale brownish tinge, yellowish in costal area and at base. Stigma distinct, oval, dark brown. Small but distinct dark spot surrounds axillary vein at wing base. Cord, distal margin of discal cell and distal longitudinal veins surrounded by indistinct brownish areas. Veins brown, yellowish at wing base. Macrotrichiae on distal veins missing. Venation: humeral vein slightly beyond arculus, Sc very long, reaching wing margin distinctly beyond r-m but before branching point of R_2+3_ and R_4_, sc-r at the level of r-m. Rs long and nearly straight, arched at base. Free end of R_1_ oblique, R_2_ and R_2+3_ equal in length. R_3_ and R_4_ diverging, cell r_3_ with long stem, which is approximately half length of Rs. Cross-vein r-m distinct, slightly oblique, in alignment with basal deflection of M_1+2_ (base of discal cell). Discal cell 1.5× longer than wide. Cross-vein m-cu very slightly beyond base of discal cell (branching point of M). Vein CuP distinctly curved at distal part, thus cell cua gets wider towards wing margin, but nearly parallel-sided from base to ~ 2/3 of its length. Anal vein long, slightly concave at middle, apex at same level as Rs base. Anal angle wide, posterior margin widely rounded. Stem of halter pale to brownish, knob dark brown. Length of male halter 1.2 mm. Coxae from brown dorsally to yellow ventrally, covered with gray pruinosity and long yellowish setae. Trochanters obscure yellow. Femur yellow with narrowly dark brown apical part. Tibia yellowish brown with narrowly darkened apex. Tibia of fore leg with single apical spur, tibiae of middle leg with two apical spurs. Tarsus brown at base, dark brown at distal end. Male femur I: 3.2 mm long, II: 4.5 mm, III: 7.5 mm, tibia I: 7.5 mm, II: 5.7 mm, tarsus I: 7.0 mm. Claw yellowish brown with subbasal spine.

***Abdomen*.** Two basal tergites brown, remaining dark brown with narrowly brownish yellow lateral margins. Three basal sternites grayish brown, remaining getting darker towards apex, lateral margins brownish to grayish yellow. Abdominal segments covered with long whitish setae, that are denser laterally. Male terminalia (Figs 52, 53) brownish, ninth segment narrower than rest of the abdomen. Epandrium wider than long, posterior margin with wide V-shaped emargination. Gonocoxite elongate, nearly 3× as long as wide at base, dorsal surface with lighter transverse area at middle which extends from less sclerotized mesal surface. Two pairs of long narrow gonostyli. Outer gonostylus sclerotized, long, slightly arched, apex distinctly narrowed and spine-shaped, mesal surface with small serration distally. Inner gonostylus elongate, fleshy and setose, apical part distinctly narrower, rounded apex. Paramere with two lobes, dorsal lobe wedge-shaped, ventral lobe elongate with rounded distal margin. Aedeagus simple, short, and straight, apex just slightly protrudes beyond frontal margin of aedeagal sheath in dorsal view. Anterior apodeme long and narrow, without lateral lobes, extending far forward.

#### Elevation range.

Ca. 20 m in Korea. 30 m to 650 m in Russia, Japan, and Mongolia (Przhiboro et al. 2009).

#### Period of activity.

Beginning of August in Korea. June–July in Russia, Japan, and Mongolia (Przhiboro et al. 2009).

#### Habitat.

Unknown in Korea. Shores of different types of running waters, from shores of large- and medium-sized rivers on plains to medium-sized and small rivers in the foothills in boreal forest, mixed forest, forest-steppe, and steppe landscape zones in Russia and Mongolia (Przhiboro et al. 2009). Both sexes are attracted to light (Przhiboro et al. 2009). Larvae of this species develop only on the bottom of large and medium sized rivers. Last instar larvae and pupae can be found in the riparian zone, usually in gravel, sand or under stones in Mongolia (Podeniene and Gelhaus 2015).

#### Distribution.

Eastern part of Russia, Mongolia and Hokkaido Island, Japan. Recorded on the Korean Peninsula for the first time.

### ﻿ Hexatoma (Eriocera) lygropis

Taxon classificationAnimaliaDipteraLimoniidae

(Alexander, 1920)

82AA5469-466B-5E65-9810-4329C1B6B6D2

Figs 54 , 56–58

#### Type material examined.

***Paratype***, male (pinned, wing slide–mounted), **China**, Formosa [Taiwan], Koshun, 25 April – 25 May 1918, J. Sonan, K. Miyake, M. Yoshino leg. (USNM).

#### Other examined material.

**Figures 54–58.**
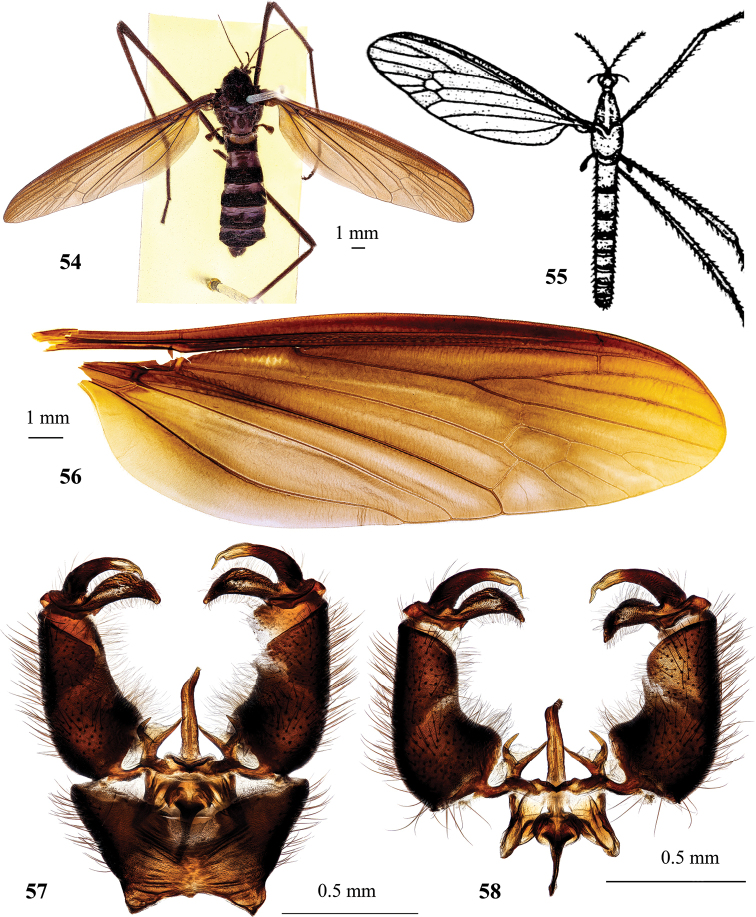
Hexatoma (Eriocera) lygropis (Alexander, 1920) **54** male, dorsal view **55** erroneous image of *H.lygropis* on which were based all Korean records **56** male wing **57** male genitalia, dorsal view **58** male genitalia with ninth segment removed, dorsal view. Scale bars: 1.0 mm (**54, 56**); 0.5 mm (**57, 58**). (**55** after Kim (1971)).

**China**, 2 males, 1 female (pinned), Formosa [Taiwan], Koshun, 25 April – 25 May 1918, J. Sonan, K. Miyake, M. Yoshino leg. (NHMUK) (Fig. 54); 1 male (pinned), Formosa [Taiwan], [Kaohsiung County – label in Chinese] (NHMUK).

#### Remark.

The first record of this species from Korea is that of Kim (1971). Unfortunately, no information was listed on which specimen(s) that record had been made, but the illustration (fig. 36) shows a species which is different from *H.lygropis* (Fig. 55). The most obvious difference is the missing cell m_1_, while *H.lygropis* has a well–developed cell m_1_. The Korean University collection, on which Kim’s (1971) publication was based, has a few *Hexatoma* specimens identified as *H.lygropis*, but all of them are in fact *H.pernigrina*, which also has no cell m_1_. All other records of that species are based only on Kim (1971). Based on this, we exclude *H.lygropis* from the Korean species list. *Hexatomalygropis* is endemic to Taiwan.

## ﻿Discussion

Crane flies belonging to the genus *Hexatoma* Latreille, 1809 (Diptera, Limoniidae) are very diverse in the Eastern Palaearctic, the fauna of which includes 72 species (Oosterbroek 2022). Despite a 90 year history of research into these crane flies on the Korean Peninsula, dating back to 1930, only three species were listed on the most recent species list of Korea (Cho 2019), this covering both North and South Korea. Due to the abundance of habitat suitable for *Hexatoma* in Korea, it was expected that more species should occur, but the genus is rather difficult and problematic taxonomically, with many Asiatic species known only from type specimens, with some of them described from females only. Male terminalia and wing venation, which are often used for discriminating species in other genera of Limnophilinae crane flies, are rather uniform in most *Hexatoma* and lack good identifying characters, thus raising the probability of misindentifications and prompting the urgent need of revision of local species at least. Based on material from all the scientific collections that were available for our study, and on our personal collecting, we were able to add six new species to the Korean species list, three of which were new to science. One species, endemic to Taiwan, was deleted from the Korean species list as a misidentification. New detailed photographs of the most important taxonomical characters and a provided identification key will be useful not only for researchers of Korean insects, but also for researchers from neighboring countries, such as China, Japan, and Russia, all of which are currently making good progress in the research into crane flies.

**Figures 59–66.**
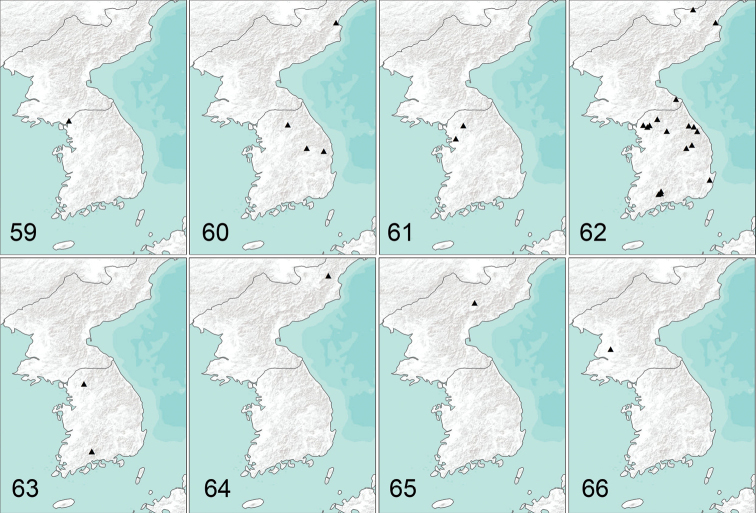
Sampling localities of Korean Hexatoma (Eriocera)**59**H. (E.) gifuensis Alexander, 1933 **60**H. (E.) ilwola sp. nov. **61**H. (E.) masakii Alexander, 1934 **62**H. (E.) pernigrina Alexander, 1938 **63**H. (E.) pianigra sp. nov. **64**H. (E.) serenensis sp. nov. **65**H. (E.) stackelbergi Alexander, 1933 **66**H. (E.) ussuriensis Alexander, 1934.

## Supplementary Material

XML Treatment for Hexatoma

XML Treatment for Hexatoma (Eriocera)

XML Treatment for Hexatoma (Eriocera) gifuensis

XML Treatment for Hexatoma (Eriocera) ilwola

XML Treatment for Hexatoma (Eriocera) masakii

XML Treatment for Hexatoma (Eriocera) pernigrina

XML Treatment for Hexatoma (Eriocera) pianigra

XML Treatment for Hexatoma (Eriocera) serenensis

XML Treatment for Hexatoma (Eriocera) stackelbergi

XML Treatment for Hexatoma (Eriocera) ussuriensis

XML Treatment for Hexatoma (Eriocera) lygropis
